# Chemometric analysis reveals links in the formation of fragrant bio-molecules during agarwood (*Aquilaria malaccensis*) and fungal interactions

**DOI:** 10.1038/srep44406

**Published:** 2017-03-14

**Authors:** Supriyo Sen, Madhusmita Dehingia, Narayan Chandra Talukdar, Mojibur Khan

**Affiliations:** 1 Biodiversity & Ecosystem Research Group, Institute of Advanced Study in Science and Technology (IASST), DST, Govt. of India, Guwahati, 781035, Assam, India

## Abstract

Fragrant agarwood, arguably the costliest wood in the world, is formed by plant-fungal interactions in *Aquilaria* spp. However, very little is known about this fragrant outcome of interaction. Therefore, mimicking the ancient traditions of agarwood production in Assam (Northeast India), a chemometric assessment of the agarwood-fungus interaction was made by chemical profiling (GC-MS) coupled with statistical analysis (principal component, correlation network analysis) across three platforms, *viz*. callus, juvenile plants and resinous wood-chips with an associated *Fusarium*. In the study of callus-fungus interaction, increased accumulation of key aroma compounds such as pentatriacontane {fold change (log2FC) = 3.47)}, 17-pentatriacontene (log2FC = 2.95), tetradecane, 2-methyl- (log2FC = 1.10) over callus and activation of pathways related to defense and secondary metabolism indicated links to aroma production. Study on fungal interactions in juvenile plants and resinous wood-chips indicated formation of terpenoid precursors (e.g. farnesol, geranylgeraniol acetate) and agarwood sesquiterpenes (e.g. agarospirol, γ-eudesmol). Correlation network analysis revealed the possible regulation of sesquiterpene biosynthesis involving squalene. Also a direct role of fungus in aroma (e.g. dodecane, 4-methyl-, tetracosane) was highlighted. Appearance of fragrant molecules unknown to agarwood during interaction featured as a new possibility for future research.

Plant based fragrances are the major constituents of commercial aroma and personal-care industry. Synthetic molecules, products of microbial fermentation and enzymatic conversion are also used apart from the traditional extraction from natural sources. However, the growing consumer preference for natural ingredients has led to renewed interest in biogenic fragrances. In this context, fragrant volatiles formed as a result of plant-microbial interactions is a potential option for the future. Biotechnological interventions including fermentation and microbial production of fragrant compounds are already being actively explored in the flavor and fragrance industry^1^,^2^,^3^. There are instances of plant-microbial associations in nature that result in formation of fragrant compounds, as in case of vetiver^4^ and agarwood. In case of agarwood (*Aquilaria* spp.; Family- Thymelaeaceae), it is well established that the fungal infection is essential for fragrant resin formation^5^,^6^,^7^,^8^,^9^. Members of a number of fungal genera comprising *Fusarium, Lasiodiplodia, Phaeoaccremonium, Melanotus*, etc., have been isolated from resinous *Aquilaria* spp. (Supplementary Figure SF-1). Chemically, resinous agarwood is a complex mixture of more than 150 compounds among which sesquiterpenes are a major determinant of its unique fragrance^10^. In the international market, agarwood drives trade worth 6–8 billion USD^11^. Natural agarwood formation is extremely rare and confined to a small geographical area. Moreover, the basic mechanism of agarwood formation is still unclear. This has seriously affected the level of R&D involved in agarwood production and is still carried out by traditional methods. Scientific studies on agarwood have mostly focused on chemical profiling of wood and oils^12^,^13^,^14^ and production of agarwood compounds by artificial induction using chemical^15^,^16^, fungal^8^,^9^ and *in vitro* cell culture interventions^17^,^18^. The efforts to understand the basic mechanism of agarwood formation so far have been focused around terpenoid biosynthesis, particularly sesquiterpenes^19^,^20^. In field, although it is believed that *A. malaccensis* plants only above the age of 10–15 years are suitable for resin formation, a recent report of artificial induction of agarwood in juvenile (4 year old) plants by fungal infection has opened new possibilities in agarwood production^9^. There is a large research need in agarwood that takes into account the diversity of interactions resulting in a complex aroma. The opportunity of comprehensive phenotype level studies (proteomics, transcriptomics and metabolomics) to understand fungus-agarwood interaction is yet to be explored. Two previous studies conducted on these aspects included transcriptomics and metabolomics analyses of healthy and resinous agarwood^21^,^22^. However, these studies did not focus on the plant-fungal interaction. Therefore, the present study for the first time uses metabolomic approaches (GC-MS, multivariate statistics and correlation network analysis) across three different platforms of interaction (involving *A. malaccensis* and an associated *Fusarium* sp.) *viz*. callus, juvenile plant and fermentation of resinous wood-chips. It aims to find chemometric signatures that can explain agarwood formation and also scan the interaction landscape for new aroma compounds. This, apart from enhancing the basic understanding of agarwood formation can potentially broaden the base of agarwood aromatics for the future.

## Results

### Isolation of associated fungi and *in vitro* callus induction

Using PDA as culture media, a total of 33 fungal associates were isolated from resinous tissue of agarwood (Supplementary Table ST-1). Simultaneously, callus from leaves of *A. malaccensis* were induced in MS medium supplemented with 3.0 μM each of 2, 4-D and BAP (Supplementary Table ST-2).

### Co-culture based screening of fungal associates

**Figure 1:**
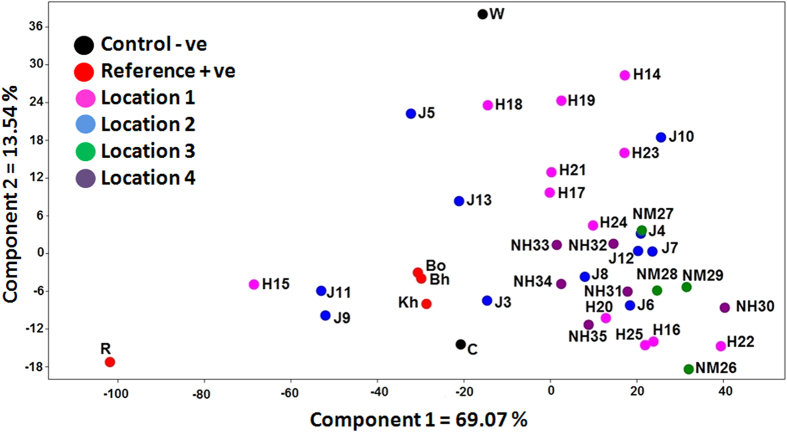
Principal component analysis (PCA) for screening of promising fungal isolates. PCA was performed with GC-MS data from co-culture of callus with 33 fungi isolated from resinous agarwood of 4 locations of Assam, *viz*. Hojai (Location 1), Janji (Location 2), Namti (Location 3) and Nahorani (Location 4). Resinous agarwood (R) and oils (*Kh, Bh, Bo*) were positive control (Reference +ve) and non-resinous wood (W) and untreated callus (C) served as negative control (Control −ve). Metabolite profiles of the fungal isolates that clustered closer to that of resinous wood and agarwood oils from the plot were subjected to ANOSIM analysis and finally 10 isolates (J3, J8, H15, NH34, J11, J9, NH31, NH35, J6 and H20) were selected as promising associated fungi of agarwood.

Fresh callus was grown in MS basal media in 24 well plates. There the 33 fungal isolates were co-cultured with the calli for a period of 12 days, during which mycelia of each isolate interacted differently to cover the calli. A PCA plot based on their GC-MS profiles is presented in Fig. 1, where the different agarwood tissues including resinous wood (R), non-resinous wood (W), uninfected callus (C) and three grades of agarwood oil, *Khara* (Kh), *Boha* (Bh) and *Boya* (Bo) are clearly distinguishable. An *a priori* set of 10 fungi, *viz*. J3, J8, H15, NH34, J11, J9, NH31, NH35, J6 and H20 were selected based upon their proximity to agarwood oil and resinous wood profiles. As expected, uninfected callus and non-resinous wood were prominent outliers. For ANOSIM analysis a total of six groups, i.e. the *a priori* assigned set (10 co-cultures), the rest of the co-cultures (23 out of 33), resinous wood, the oils, non-resinous wood and uninfected callus were assigned. The results confirmed that the *a priori* group of 10 co-cultures differs significantly from the group comprising rest of the 23 co-cultures (R = 0.326; *p* = 0.004) but was similar to the resinous wood (R = 0.995; *p* = 0.092) and oils (R = 0.195; *p* = 0.122). At the same time, the group consisting the rest 23 co-cultures was significantly different from the resinous wood (R = 1.0; *p* = 0.043) and oils (R = 0.95; *p* = 0.0009).

### *In vitro* simulation of agarwood fermentation

The 10 best fungal isolates were freshly co-cultured with agarwood callus (12 days) and subsequently fermented *in vitro* for 30 days. H15 (NCBI Acc. No. KP000001), identified to be a *Fusarium* by ITS sequencing, was selected based on superior growth, attachment and viability after 20 days of fermentation with callus and formation of aroma compounds such as spiro [4.5] dec-7-ene, 1, 8-dimethyl-4-(1-methylethenyl)-, [1S-(1α, 4β, 5α)]- (CAS No. 24048-44-0). An Indian patent (No. 1277/KOL/2014) has been filed for *in vitro* production of fragrant agarwood based on the findings^23^.

### Interaction of *A. malaccensis* callus with associated *Fusarium* isolate (strain H15)

**Figure 2:**
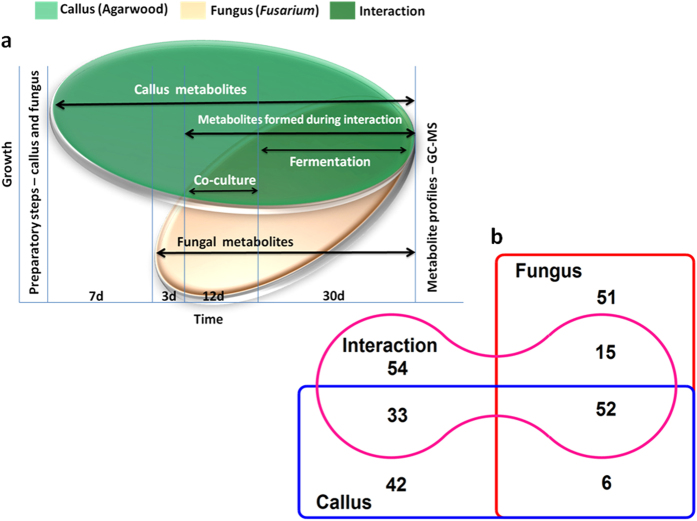
Chemometric analysis of the interaction between agarwood callus with a promising *Fusarium* associate. Agarwood callus (5.0 g) was co-cultured with *Fusarium* (400 μl in PDB) for 12 days followed by fermentation in sterile water (40 ml) in Erlenmeyer flasks (incubated in dark at 25 ± 2 °C) for 30 days. Pure cultures of callus and *Fusarium* grown identically served as experimental controls. At the end of the 52 days experiment, the terminal metabolite profiles of pure callus, pure *Fusarium* and callus-*Fusarium* interaction were determined from GC-MS of their n-hexane extracts (**a**). The Venn diagram represents the distribution of a total of 253 compounds in callus, fungus and interaction (**b**).

Evan’s test showed that *A. malaccensis* callus remained viable till at least a month of co-culture with an associated *Fusarium* strain H15 (Supplementary Figure SF-2). Based on the findings an experiment as represented in Fig. 2a was performed. GC-MS followed by Venn analysis revealed a catalogue of 253 compounds distributed among the 3 profiles *viz*. callus, fungus and interaction. A total of 51, 42 and 54 unique compounds were present in fungus, callus and interaction, respectively. Among the 3 profiles, 52 compounds were common; 15 compounds were shared between fungus and interaction, 33 between callus and interaction and 6 between callus and fungus (Fig. 2b). Further details are as follows:

#### Compounds unique to interaction

The highest number of unique compounds (54) was identified in the interaction profile. Among them 36 compounds have been previously reported in essential oils and aroma compounds of plant and fungal origin and 11 compounds were reported in fragrant profiles of agarwood (see Supplementary Figure SF-3 and Supplementary Table ST-3). The metabolite profile was dominated by alkanes and alkenes (17) followed by esters (12) and also acids including fatty acids (6), alcohols (4), aldehydes and ketones (5), acetates (3), terpenes (2) and other aromatic/volatile compounds (5).

#### Compounds shared by callus and interaction

The profiles of callus and interaction contained 33 common compounds. Fold change analysis classified the compounds into following three groups (see Supplementary Table ST-4.1):

- Compounds that decreased during interaction (log2 FC < 0.00) - 6 compounds,
- Compounds that showed major increase during interaction (log2 FC > 1.0) - 13 compounds, and
- Compounds that showed moderate to no increase during interaction (log2 FC 0.00–1.00) - 14 compounds.

**Figure 3:**
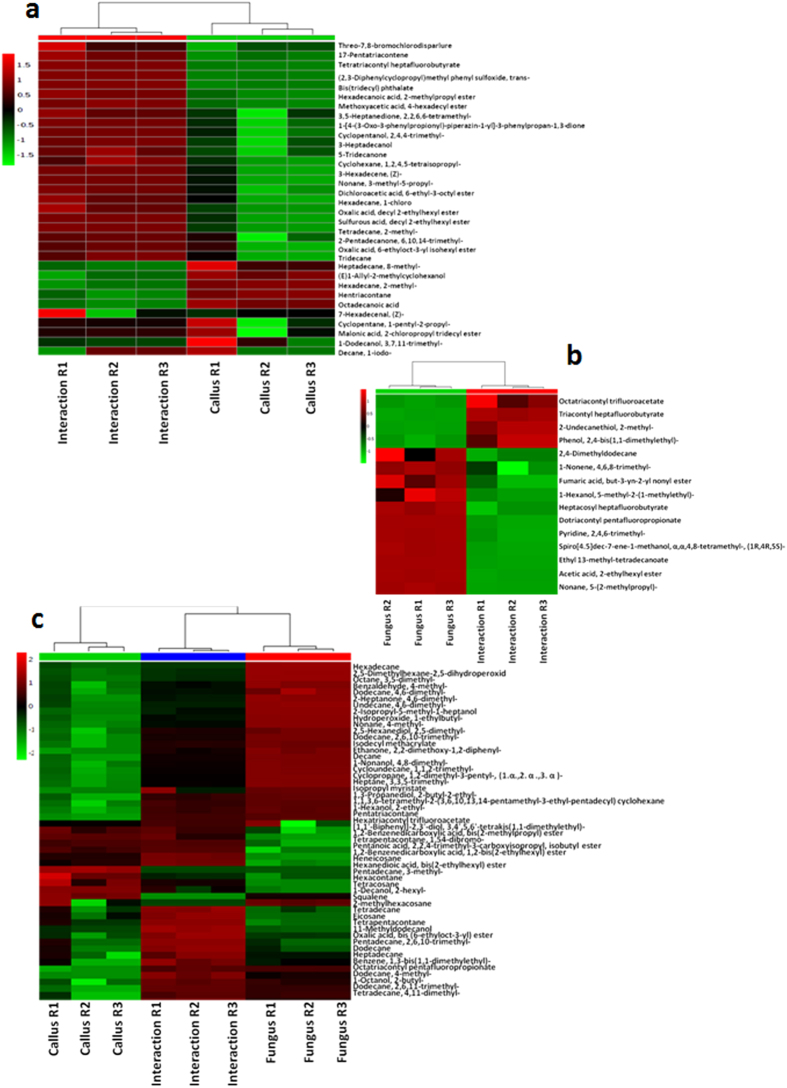
Heat map analysis of the metabolites of callus-fungus interactions. Perturbations in levels of accumulation of the 33 compounds shared by profiles of callus and interaction (**a**), 15 compounds common to fungus and interaction (**b**) and 52 compounds shared by the profiles of callus, fungus and interaction (**c**) based on their relative peak area (%).

Figure 3a and Supplementary Tables ST-4.2 and ST-4.3 present details of the compounds. Out of the 6 compounds that showed substantial decrease during interaction, 5 were reported in aromatic profiles of plants and 3 compounds (octadecanoic acid, 1-dodecanol, 3, 7, 11-trimethyl- and hentriacontane) in agarwood. Hexadecane, 2-methyl-, heptadecane, 8-methyl- and hentriacontane are important constituent of plant epicuticular wax. Among the 13 compounds that showed major increase, 2 compounds (17-pentatriacontene and tetradecane, 2-methyl-) are important aroma component of agarwood. Majority of the compounds are alkanes/alkenes (5) followed by esters (3), ketones (2), alcohols (1) and others (2).

#### Compounds shared by fungus and interaction

Metabolite profiles of fungus and interaction shared 15 compounds. Fold change analysis revealed that level of accumulation of 11 compounds decreased during interaction while the remaining 4 increased (Supplementary Table ST-5.1). Figure 3b and Supplementary Tables ST-5.2 and ST-5.3 present the details of the compounds. Out of the 11 compounds, acid esters (5) and alkanes/alkenes (3) were the major chemical classes. Out of these, 3 compounds (acetic acid, 2-ethylhexyl ester, pyridine, 2,4,6-trimethyl- and alpha-acorenol) are components of fragrant profiles previously reported in fungi including *Fusarium.* Apart from that 4 fungal compounds increased during interaction, consisted of two prominent halogenated acid esters triacontyl heptafluorobutyrate and octatriacontyl trifluoroacetate (log2 FC = 0.62–1.25).

#### Compounds shared by the three profiles - callus, fungus and interaction

The shared profiles of callus, fungus and interaction had 52 common compounds. The perturbation in the level of metabolites is shown in Fig. 3c. Fold change analysis was performed separately for interaction/callus as well as interaction/fungus and comparative levels of change *vis-à-vis* callus and fungus profiles was ascertained (Supplementary Table ST-6). The profile was dominated by alkanes/alkenes (30) followed by esters (9) and alcohols (7). It was observed that 17 out of 52 compounds (32.69%) were previously reported in agarwood. Of the 17 agarwood specific compounds 16 were reported in plant essential oils and fungal profiles. Fold change analysis for interaction/callus revealed that level of accumulation in 44 out of the 52 compounds (i.e. 84.62%) increased during interaction within the range of log2FC 0.05 to 3.64, while level of 8 compounds (15.38%) decreased during interaction within the range of log2FC −0.01to −6.71. In case of interaction/fungus 26 compounds (50.0%) showed increased formation within the range of log2FC 0.068 to 2.87 while the rest 50.0% decreased in the range of log2FC −0.23 to −4.07. In total, 28 compounds were reported in global aromatic (and essential oil) and fungal profiles that included *Fusarium* and 15 of them were reported in agarwood. The level of accumulation of 15 compounds increased against both callus as well as fungus during interaction. The highest fold change was tetrapentacontane, 1, 54, dibromo-(Log2FC interaction/callus = 2.50; Log2FC interaction/fungus = 2.87) which is a constituent of the fragrant profile of several essential oils. Among the agarwood specific compounds that enhanced during interaction, pentatriacontane showed the highest increment (log2FC = 3.471) over callus with a marginal reduction over fungus (log2FC = −0.385). Similar changes were observed in case of dodecane, 4,6-dimethyl-, 1-nonanol, 4,8-dimethyl-, hexadecane (highest reduction over fungus; log2FC = −4.07), isopropyl myristate and 1-hexanol, 2-ethyl-.

#### Compounds shared by callus and fungus and compounds unique to callus and fungus

A total of 93 compounds out of 253 (36.75%) were unique in profiles of callus (42) and fungus (51). There were additionally 6 compounds, common to fungus and callus. The details are presented in Supplementary Results section and Supplementary Tables ST-7, 8 and 9.

### Metabolite profile of juvenile *A. malaccensis* plants infected with associated *Fusarium*

**Figure 4:**
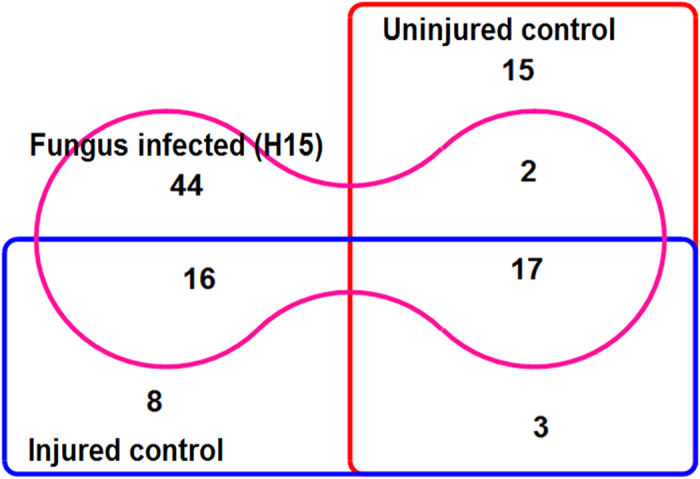
Juvenile agarwood plants artificially infected with *Fusarium* associate. *Fusarium* culture was added @ 10.0% (v/v) into a broth consisting of 2.0% (w/v) sucrose and 0.01% (v/v) Tween20 and injected (1.0 ml) into holes made in the stem of juvenile agarwood plants during rainy season. After injection the wound was covered with sterilized cotton and secured with cling film. The holes were re-inoculated twice and kept moistened regularly with sterile water. At the end of 3 months the distribution of compounds detected in the GC-MS profiles of un-injured control, injured control and *Fusarium* fungus infected tissue extracts are represented in the Venn diagram.

Juvenile agarwood plants were injured by drilling holes and then infected with *Fusarium* strain H15 and the site of inoculation was kept covered for a period of 3 months. The zone of discoloration around the holes after 3 months appeared similar to resinous wood. Although fungus treated agarwood plants showed higher area of discoloration, it was not significantly higher than control and neither was the Pearson’s correlation between girth of the plant and area of discoloration. Therefore to determine the role of fungal infection in the changes observed, a comparison of their metabolite profiles was performed. GC-MS revealed a total of 105 compounds distributed among 3 distinct tissue types *viz*. uninjured control (Control-Nh), injured control (Control-H) and fungus infected (H15) (Fig. 4). Interestingly, the highest numbers of compounds (44) was formed only during infection by fungus. A detailed analysis of the compounds formed only during interaction between fungus and juvenile agarwood revealed 11 compounds (25.0%) previously reported in resinous agarwood. The profile included 12 esters (27.27%), components of essential oils such as benzeneacetic acid, 3-tridecyl ester, undecanal and also included important semio-chemicals and precursors of fragrant molecules (farnesol, methoprene, geranylgeraniol acetate). The details can be found in Supplementary Table ST-10.

### Metabolite profile of fermentation of resinous agarwood chips with associated *Fusarium*

**Figure 5:**
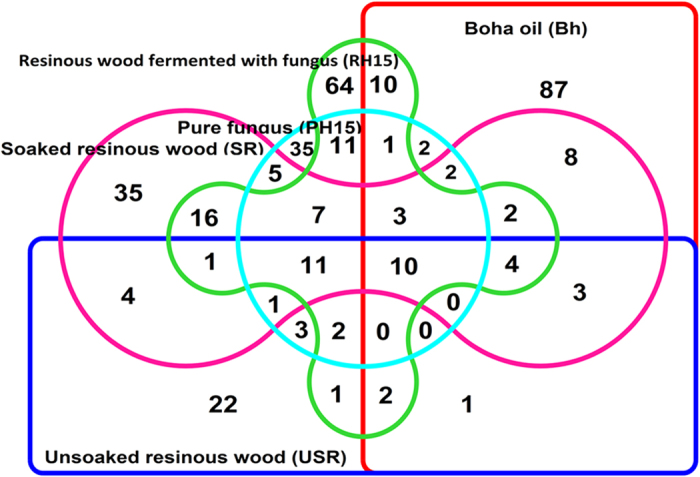
Fermentation of resinous agarwood chips with promising *Fusarium*. Sterile 50 ml tubes containing 10.0 ml sterile water were filled with 1.0 g resinous agarwood chips (pulverized, surface sterilized). Fresh culture of *Fusarium* was added into the tubes and incubated under ambient conditions for 45 days. Pure cultures of *Fusarium* and resinous wood alone served as control in the soaking experiment, apart from un-soaked resinous wood. *Boha* agarwood oil was included as reference for comparing the GC-MS profiles. The Venn diagram shows a distribution of 353 compounds across the 5 profiles. Interestingly the profile of fermentation of chips with *Fusarium* strain H15 included major agarwood aroma constituents such as aristolene epoxide, γ –eudesmol, agarospirol, (−)-aristolene, (−)-globulol, 6-(1-hydroxymethylvinyl)-4,8a-dimethyl-3,5,6,7,8,8a-hexahydro-1H-naphthalen-2-one, isophytol, cyclotetradecane, 1,7,11-trimethyl-4-(1-methylethyl)-, cyclohexane, 1,4-didecyl-, propanoic acid, 3,3′-thiobis- didodecyl ester.

Resinous agarwood chips were fermented for a period of 45 days with the promising *Fusarium* stain H15. A total of 353 compounds were identified in the 5 profiles, *viz. Boha* oil (Bh), unsoaked resinous wood (USR), soaked resinous wood (SR), resinous wood fermented with fungus (RH15), and pure culture of fungus fermented without resinous wood (PH15). Venn analysis (Fig. 5) determined the distribution of the compounds revealing the second highest number of unique compounds in RH15 (64) behind *Boha* oil (87). A total of 78 unique compounds were formed in SR that were absent in USR and 91 compounds occurred in RH15 which were absent in SR. However, most interestingly, among the 10 compounds common between *Boha* oil (Bh) and resinous wood fermented with fungus (RH15), key agarwood sesquiterpenes (e.g. γ-eudesmol, agarospirol, aristolene) were formed only during fermentation of the resinous agarwood chips with *Fusarium*. These compounds were absent in the rest of the profiles which confirms a role of fungus during the fermentation. Considering the future application of the findings in the aroma industry an Indian patent of addition (No. 201633016084) has been filed^24^.

### Correlation network analysis

**Figure 6:**
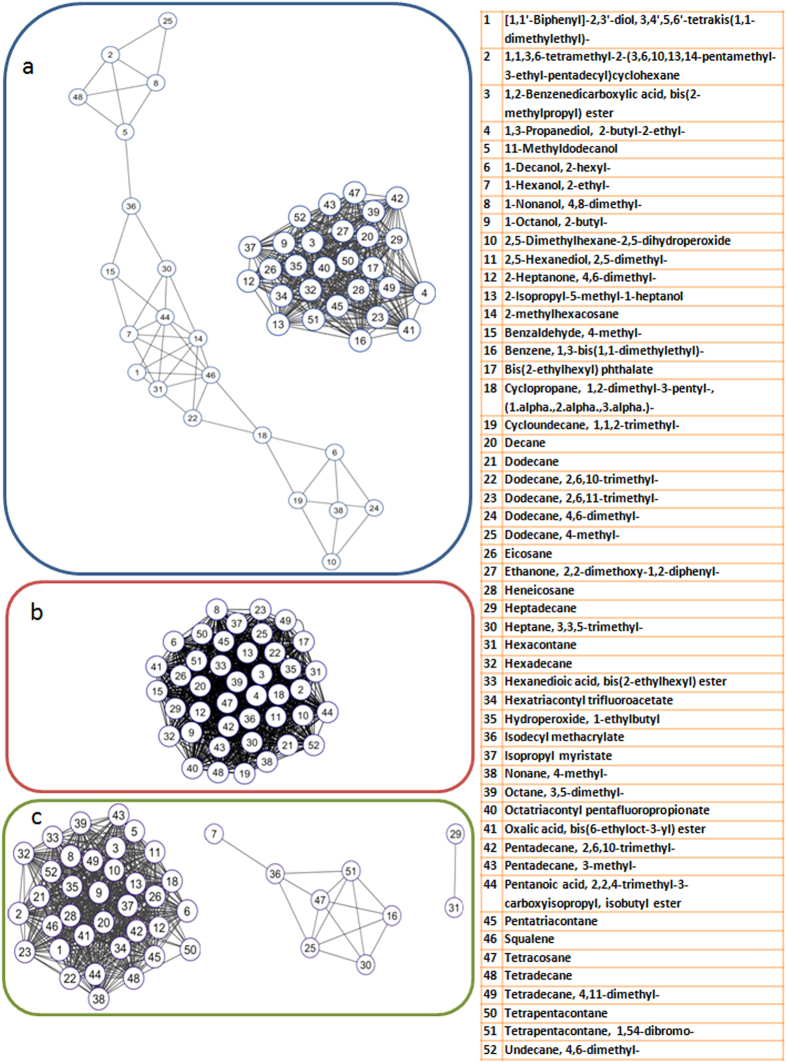
Correlation network analysis. Network topology of the 52 compounds shared by the metabolite profiles of callus, fungus and interaction were different. In case of the fungus network (**a**) the 42 correlated compounds were clustered within a single network, whereas the callus network (**b**) comprised of two independent networks of which 49 compounds were correlated and the majority (28) were clustered in a dense plot. The rest (21) compounds were in a group of 3 smaller networks of 5, 6 and 10 nodes. In case of interaction network (**c**) a significantly different topology was observed. 44 compounds formed 3 independent networks, one being a dense cluster of 35 compounds while the other two comprised of 2 and 7 compounds respectively.

Correlation network analysis was attempted to simplify the complex picture. Correlation between the 52 compounds shared by the profiles of callus, fungus and interaction when plotted separately as networks generated clear-cut differences in network topologies (Fig. 6). A closer scrutiny of the networks based upon the findings from the metabolite profiles (callus, juvenile plants and fermentation of chips) was performed by studying the correlation pattern (partners and nature of correlation) of few important compounds. In case of squalene, the number of significant correlations increased from 0 (fungus) and 7 (callus) up to 27 (interaction) as its level decreased over callus and fungus. Due to such changes the network topology was altered to accommodate newer correlations. Interestingly, squalene demonstrated negative correlations with 15 compounds only during interaction. Similarly, in case of tetrapentacontane 1, 54-dibromo-, the number of correlations decreased from 29 (fungus) and 17 (callus) to 5 (interaction) as the concentration of the compound increased during interaction and as a consequence the network topology changed. A closer look at the pattern revealed its consistently positive correlation with tetracosane, an important agarwood compound in callus, fungus as well as interaction. However, correlation profile of tetracosane revealed 5 interactions which included positive correlation with dodecane, 4-methyl, which is another important fragrant molecule. Apart from this, a similar pattern of change in the network topologies for compounds shared by callus and interaction as well as fungus and interaction (Supplementary Figures SF-4 and SF-5) clearly indicate substantial metabolic modifications induced by interaction.

## Discussion

### *Fusarium* and agarwood

Metabolite profiling and multivariate statistical tools were able to distinguish chemically significant interactions and identify promising fungal associates. The most promising isolate (H15) was found to belong to the genus *Fusarium*. In fact the members of the genus *Fusarium* are a key fungal associate of agarwood (Supplementary Figure SF-1). In case of agarwood, interaction with *Fusarium* as a pathogenic challenge and a defensive counter-response appears to have been favoured in nature towards resin formation. *Fusarium* – plant interaction, particularly the infection machinery of the fungus involving penetration and colonisation of the plant tissue with the help of fungal secondary metabolites (mycotoxins) have evolved during co-existence^25^. The plant responds by chemical defence. Terpene containing resins which form a component of such defence can restrict the proliferation of mycelia and kill fungal spores^26^.

### Interactions of *A. malaccensis* callus with associated *Fusarium* (H15)

#### Compounds unique to interaction

Among the compounds unique to the interaction profile, n-hexadecanoic acid (palmitic acid) was the most abundant (Supplementary Table ST-3 and Supplementary Figure SF-3). Biotic challenge such as fungal infection often leads immediately to the increased formation of free fatty acids that trigger oxidative burst and fatty acid oxidation cascades leading to production of oxylipins such as jasmonates^27^,^28^. Accumulation of palmitic acid also signals the beginning of lipid biosynthesis from carbohydrate precursors. Octadecanoic acid ethyl ester is an ester of octadecanoic acid (stearic acid). It was observed that free stearic acid content had substantially reduced during interaction as a possible result of the fragrant ester formation. In apple, decrease in concentrations of free fatty acids such as palmitic, linolenic, oleic, linoleic, stearic acids has been associated with the increase of aroma production^29^. An important fatty acid 9, 12-octadecadienoic acid (Z, Z)- or linoleic acid was formed during interaction. Linoleic and linolenic acid are the precursors for lipoxygenase (LOX) that initiates the octadecanoate and lipoxygenase pathways responsible for the formation of jasmonates and leaf volatiles^30^. Among the terpenes, geranyl isovalerate with its fruity aroma is an important semio-chemical (pheromone). Reported both from *Aquilaria* as well as *Fusarium*, it assumes significance as insect infestation can predispose agarwood to fungal infection. Among the known agarwood compounds (9), a majority (4) were alkanes. Alkane biosynthesis in plants although poorly understood is important to stress response, particularly in the formation of cuticular wax. Wax forms as an acyl chain by the condensation-elongation of acetyl-CoA and malonyl-CoA. The acyl chain is subsequently modified to include 1° and 2° alcohols, aldehydes, ketones, esters and terpenoids apart from long chain fatty acids^31^. In the present study, formation of alkanes (e.g. 2-methyltetracosane, triacontane-1, 30- dibromo), aldehydes (e.g. E-15- heptadecanal), alcohols, esters and other compounds (e.g. malonic acid, 2-methylpentyl undecyl ester) were notable. The contribution of fungus to the fragrant profile is remarkable as seen in geranyl isovalerate (from *Aquilaria* as well as *Fusarium*), cyclohexanol, 4-methyl-1-(1-methylethyl)-, benzoic acid, 4-methyl-, i-propyl 12- methyltetradecanoate, tetradecanal and pentadecane, 4-methyl-. Also the effect of fermentation can be seen from accumulation of compounds such as fumaric acid esters that are known to be formed during submerged fermentation^32^.

#### Compounds shared by callus and interaction

The compounds that decreased during interaction were mostly alkanes (Supplementary Table ST-4 and Fig. 3a). Hexadecane, 2-methyl-, heptadecane, 8-methyl- and hentriacontane, are constituents of the protective wax layer in plants. The reduced level of wax is therefore an apparent indication of infection. However, the perception of cuticular wax by fungus and the host plant is very complex^33^. Fungal enzymes such as cutinases are responsible for the breakdown of cuticular components such as waxes to facilitate penetration. In early infection, the enzymatic breakdown products of cuticle become the inherent cellular signal perceived by the plant which plays the role of initial elicitor of a defense response. Interestingly, at pre-penetration stage the production of such enzymes (cutinases) by the fungus is activated by the perception of the cuticular wax on the plant cell surface itself. Therefore, onset of pathogenic interaction can be linked to the reduction in cuticular wax content of the callus. On the other hand, phthalates that are important perfume ingredients including agarwood showed increase in level. Other compounds that increased were 17-pentatriacontene (an important constituent of agarwood oil), 3-heptadecanol (a perfume fixative) and tetradecane, 2, methyl-. Of the 14 compounds that showed moderate to low increase, tridecane (wax) is an elicitor of jasmonate and salicylic acid pathways in *Arabidopsis*^34^. Conversion of palmitic acid by esterification was the likely reason for palmitic acid isobutyl ester formation. The compounds such as hexahydrofarnesyl acetone enhanced the possibility of formation of aromatic compounds by availability of substrate. Increase in content of oxalic acid esters is of interest since pathogenesis and oxalate secretion by fungi are correlated and also of their role in lignocellulosic degradation might be linked to agarwood formation^35^.

#### Compounds shared by fungus and interaction

Fungi are known to form unusual secondary products such as halogenated acids during stress^36^. Therefore increased production of fluorinated acid esters is an indication of secondary product formation (Supplementary Table ST-5, Fig. 3b). Phenol, 2,4-bis(1,1-dimethylethyl)- was reported previously in agarwood aromatics and is also a component of several fungal aromatic profiles including *Fusarium*. This reinforces the view that the fungal associate undergoes considerable physiological modifications during interaction.

#### Compounds shared by all three: callus, fungus and interaction

The core metabolite profile showed that apart from the plant, the fungal associate contributes substantially (Supplementary Table ST-6 and Fig. 3c). Among the core set of 52 compounds, 17 (32.69%) were reported in agarwood as against only 9 out of 54 compounds (16.67%) in interaction. The frequency was still lower in case of other profiles in the present study. It was further noted that 16 out of the 17 known agarwood compounds were also present in profiles of both plant essential oils and fungi, which further points to the combined role of fungal and plant metabolites in the final agarwood aroma. Tetrapentacontane, 1,54, dibromo-, a constituent of fragrant essential oils and pentatriacontane, an important agarwood component increased considerably. Accumulation of compounds such as 11-methyldodecanol and heptane, 3,3,5-trimethyl- in relatively high concentration during interaction suggests lipid oxidation^37^. Squalene, a triterpene showed an overall reduction over both callus and fungus during interaction. Vogeli and Chappell^38^ have shown in fungal elicitor treated tobacco suspension culture that the conversion of isoprenoid intermediates (farnesyl diphosphate) into sesquiterpenes was linked to an increase in sesquiterpene cyclase activity and a co-ordinated decrease in the squalene synthetase activity obviously to channelize the flux towards sesquiterpene instead of triterpene.

In the profile of interaction although important agarwood aroma compounds were formed or enhanced, the key agarwood sesquiterpenes were absent. This indicates a possibility of regulation of sesquiterpene biosynthesis at metabolic check-points. Transcriptomic analysis on a time-scale manner would likely reveal such regulation.

### Infection of juvenile *A. malaccensis* plants and fermentation of resinous agarwood chips with associated *Fusarium* (H15)

Natural agarwood formation is considered to be influenced by age of plant (more with maturity) and season (wet higher than dry). The inherent softness of agarwood tissue and the predominantly wet and humid climate prevailing in the South and Southeast Asia where agarwood is found, provide a unique environment for prolonged fungal contact leading to infection and fermentation. The fermentation of resinous chips with fungus provided the opportunity to understand another instance of microbial involvement with regard to agarwood production. In case of juvenile plants (Supplementary Table ST-10 and Fig. 4), appearance of 9, 12-octadecadienoic acid (Z, Z)- (linoleic acid) in injured control (Control-H) and fungus infected plants (H15) bear indication of the perception of threat and response due to fungal attack. Squalene, heneicosane, bis(2-ethylhexyl) phthalate, dodecane, heptadecane and tetradecane were detected in all juvenile tissues including plants not injured or inoculated with fungus. Squalene was detected in all juvenile tissues, but was absent in terpenoid rich resinous chips indicating that regulation of sesquiterpene biosynthesis in agarwood might include squalene. From the distribution of metabolites in GC-MS profile of the fungus mediated fermentation of resinous agarwood chips (Fig. 5), the most significant finding was the appearance of key agarwood sesquiterpenes such as agarospirol, γ-eudesmol, (−)-aristolene exclusively during fermentation with *Fusarium* (H15). This indicates a major role of fermentation in determining the aroma content of agarwood reported in our previous study^22^. Fundamentally, fungus-callus and fungus-juvenile plant interaction are different from fermentation of chips with fungus as the resinous wood chips are dead tissue. Agarwood sesquiterpene are predominantly of plant origin. Therefore, the findings in the present study may be either because, a. fermentation of resinous wood provided unique substrates for fungal cellular/enzymatic machinery possibly involving biotransformation that led to appearance of aroma compounds including sesquiterpenes; b. fungal enzymatic machinery was activated during fermentation that led to increased release of sesquiterpenes from resinous mass embedded in the wood tissue and/or fungal sesquiterpene synthases were activated that led to formation of sesquiterpenes. There is an evidence of *Fusarium* spp. secreting sesquiterpenes during plant-microbe interaction leading to morpho-physiological changes in the plant^39^. Therefore, the follow up for these findings would be to establish the mechanism of fermentation assisted appearance of sesquiterpenes in the extracts. For this, intense time-scale studies would be necessary to dissect the fermentation process at different levels of phenotype (transcriptome, proteome and metabolome).

### Correlation network analysis

The complexity of the interaction between fungus and agarwood was reflected in the perturbation of metabolites studied over three different platforms. Interpretation of such complex and large-scale datasets is challenging and therefore network visualisation of correlation can provide an unbiased analysis with application to untargeted metabolomics where the pair-wise correlations reflected the physiological state of the metabolite phenotypes in question^40^,^41^. The clear cut difference in network topology (shape and distribution) provided the initial estimate of the definite interaction between fungus and callus (Fig. 6). The final concentration of a metabolite during interaction was likely influenced by suppression (decrease over callus as well as fungus, e.g. squalene), elicitation (major increase over callus, e.g. pentatriacontane) or complementation (major increase over fungus; e.g. tetrapentacontane, 1,54-dibromo-). In correlation networks, strong metabolite correlations may or may not mean that the compounds are neighbours in actual metabolic networks and pathways^42^. Therefore a pragmatic approach was adopted in comparing the networks. In case of squlaene, absence of correlation in fungus and the appearance of 15 fresh negative correlations during interaction is interesting considering that a negative relation exists in the biosynthesis of sesquiterpenes and triterpenes (squalene). The newly observed negative correlations also included resinous agarwood compounds such as pentadecane, 2,6,10-trimethyl-, isopropyl myristate, 11-methyldodecanol and pentatriacontane which suggest a strong possibility of metabolite shift towards agarwood formation with decrease in squalene. Tetrapentacontane, 1, 54-dibromo- content increased during interaction and showed positive correlation with tetracosane, and dodecane, 4-methyl which are fragrant molecules. Scrutiny of the networks revealed that correlation exists in fungus and interaction but not in callus which further points at a possible fungal influence (tetracosane and dodecane, 4-methyl). In fermentation, dodecane, 4-methyl has been detected in soaked resinous wood (SR), resinous wood fermented with the fungal strain H15 as well as in the pure fungal strain while tetracosane formed only during fermentation of resinous wood with the fungus. Therefore correlation network analysis was able to indicate for the first time a definite possibility of fungal contribution in formation of important agarwood compounds.

### The fragrant outcome of interaction

**Figure 7:**
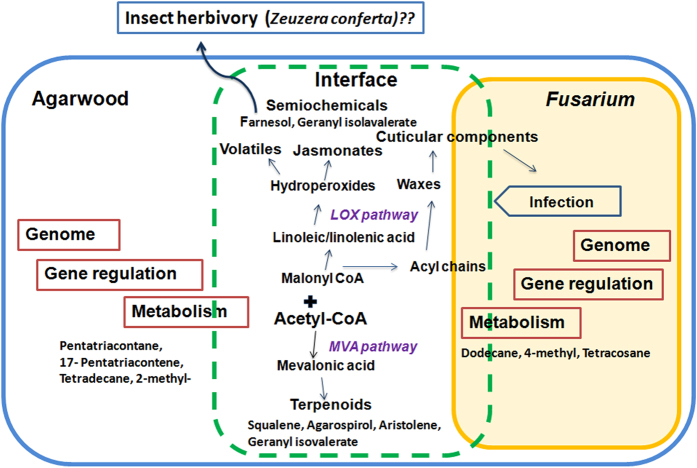
Fragrant agarwood as an outcome of fungus-plant interactions. The layout of plant–pathogen interaction given in Pinney (2011)^43^ was adopted to summarize the findings. The complex regulation of the genetic and metabolic machineries of agarwood and *Fusarium* at the interface led to modulation of defense, secondary metabolism related pathways (LOX, jasmonate, waxes, MVA) and metabolites. The comparison of metabolic phenotypes by GC-MS based profiling and correlation network analysis, revealed change in levels of terpenoids, waxes, volatiles, semiochemicals and other key metabolites (agarwood sesquiterpenes) that bear chemometric signatures of fragrant agarwood production.

The fragrant outcome of plant-microbial interaction in agarwood is an extremely fascinating phenomenon. Agarwood production is confined to a few parts of South and Southeast Asia where humid climate is prevalent that encourages fungal growth. Agarwood tissue is soft by nature. Therefore, the injuries that are easily inflicted by herbivores, predispose genetically susceptible agarwood plants to prolonged fungal infection and fermentation leading to quality agarwood production. The depiction of the complexity of this association, established over centuries of co-evolution is presented in Fig. 7, adapted from the analogous host-pathogen interaction models^43^. In the present study, *Fusarium-* agarwood interaction indicated typical perception-response mechanisms seen in biotic stress response involving waxes and cuticular barriers^33^, cellular dynamics of free fatty acids leading to oxidative burst and activation of the lipoxygenase (LOX) pathway^28^. However, from the point of view of aroma, fungal interaction between callus and juvenile plants resulted in fewer terpenoids compared to the fermentation of resinous agarwood chips. Wound associated signals have been found to be critical pre-requisites for strong expression of sesquiterpene synthase genes. In a recent study^44^ the administration of heat shock (imitating the burn-chisel-drill method practiced in agarwood trees) on *A. sinensis* suspension cultures was found to induce jasmonate signaling that led to formation of agarwood sesquiterpenes. Acetyl CoA is at the check-point of primary and secondary metabolism. Wax biosynthesis, LOX (jasmonate pathway) and MVA pathway start at acetyl CoA. Therefore the nature of regulation at this point and at the beginning of MVA and MEP pathways (and their cross talk) during *Aquilaria* –fungus interaction determines the formation of sesquiterpenes. A detailed time-point study on formation of key metabolites is necessary (e.g. levels of agarospirol, squalene, etc). The reason key agarwood sesquiterpenes were not detected during *in vitro* callus-fungus interaction is likely to be revealed by such studies. Nevertheless, the study has for the first time brought into focus the contribution of fungal metabolites (e.g. dodecane, 4-methyl, tetracosane) and non-terpene constituents like esters, alcohols towards the unique aroma of agarwood (e.g. 3-heptadecanol that confers fixative property). Further, from the *in vitro* co-culture and fermentation the fragrant molecules hitherto unknown to agarwood aroma were accumulated. This offers a unique and exciting possibility for developing newer agarwood based aroma profiles that can be commercially exploited. From the mechanistic point of view, the appearance of ecologically important semio-chemicals (e.g. pheromones) during interaction pointed to an important aspect of agarwood production. The involvement of insect borer (*Zeuzera conferta*) is well known in case of Assam agarwood. The borer tunnels into the agarwood stem as it chews up the wood following which dark resinous deposits appear along the path taken by the borer^45^. Whether semio-chemicals have a role in an insect-mediated fungal colonization of the inner agarwood tissue (heartwood) is an important question that requires proper study. Understanding the biotic interactions (plant-fungus-insect) can strengthen research on newer biogenic fragrances as well as answer fundamental questions on the complex regulation of the metabolic pathways during interaction resulting in aroma compound formation.

## Methods

### Agarwood samples

Agarwood samples (resinous and non-resinous wood, agarwood oils, etc.) were collected from four major agarwood production sites of Assam in Northeast India *viz*. Janji (26°52′N; 94°27′E), Hojai (26° 0′18′N; 92°51′E), Namti (26°51′N; 94°37′E) and Nahorani (26°13′N; 93°52′E) that are known globally for high quality agarwood.

### Isolation of associated fungi and *in vitro* callus induction

Fungi associated with *A. malaccensis* were isolated by following conventional spread plate technique over potato dextrose agar (PDA) medium. Simultaneously, callus from tender leaf tissues of *A. malaccensis* were induced in modified Murashige and Skoog (MS) medium. Refer Supplementary Methods for details.

### Co-culture based screening for fungal associates

The fungal isolates (33 nos.) were revived in potato dextrose broth (PDB). Separately, 1.0 g (fw) of one month old *A. malaccensis* calli were aseptically transferred into sterile 24 well plates, containing 2.0 ml MS basal medium per well. After 3 days, 20 μl of suspension of each of the 33 fungal isolates was added to the callus and incubated under dark at 25 ± 2 °C for a period of 12 days. n-Hexane extract of fungus-callus co-culture along with reference agarwood material *viz*. resinous wood (R), commercial grades of raw Assam agarwood oils [*Khara (*Kh), *Boha (*Bh) and *Boya* (Bo)] and negative controls *viz*. uninfected callus (C) and non-resinous agarwood (W) were subjected to qualitative GC-MS analysis. Principal component analysis (PCA) and analysis of similarity (ANOSIM) were performed in Past 2.7c software to determine chemometric relationships among the metabolic phenotypes.

### Simulation of agarwood fermentation

Out of the 33 isolates, 10 fungi were screened out (*viz*. J3, J8, H15, NH34, J11, J9, NH31, NH35, J6 and H20) and used to mimic the traditional fermentation of resinous agarwood as is practiced in Assam. To simulate the process in laboratory, fresh culture of fungus (400 μl of 3 day old culture in PDB) was co-inoculated onto Petri plates containing *A. malaccensis* callus (5.0 g) in MS basal medium and incubated in dark for 12 days at 25 ± 2 °C. The contents were thereafter transferred into 100 ml Erlenmeyer flasks containing 40 ml sterile water and fermented in dark at 25 ± 2 °C for another 30 days. GC-MS profiles of their n-hexane extracts were compared for presence of aroma compounds.

### Identification of fungi by ITS sequencing

Total DNA was extracted from actively growing fungal mycelia^46^. This was followed by amplification and sequencing of ITS rDNA using ITS1-ITS4 primer pair. Please refer Supplementary Methods for details.

### Interaction of *A. malaccensis* callus with associated *Fusarium*

A preliminary assessment of the nature of interaction of agarwood callus with the associated *Fusarium* (H15) was carried out by dual culture. Evaluation of cell viability, as the fungal mycelia covered the callus, was done by Evan’s test^47^. Thereafter, a detailed chemometric study of the interaction between agarwood callus and its associated *Fusarium*, was performed (Fig. 2a). The total experimental period was 52 days. Fresh callus (5.0 g) was cultured on each MS basal plate for 7 days. On the 7th day *Fusarium* (strain H15) was separately grown in PDB and on 10th day the fungal culture (400 μl) was added onto the plates containing callus and incubated under dark at 25 ± 2 °C for 12 days. The contents were subsequently fermented in sterile water (40 ml in 100 ml Erlenmeyer flasks incubated in dark at 25 ± 2 °C) for another 30 days. The effective period of fungus-callus interaction therefore was 42 days. Separately grown pure cultures of callus (5.0 g) and *Fusarium* (400 μl) were subjected to the same steps to serve as experimental controls. At the end of the experiment n-hexane extracts of fungus-callus co-culture (interaction), callus only (callus) and *Fusarium* only (fungus) were analyzed by GC-MS.

### Infection of juvenile *A. malaccensis* plants with associated *Fusarium* (Strain H15)

Healthy two year old *A. malaccensis* plants growing in the experimental agarwood plantation of the institute (IASST, Guwahati) were injected with the associated *Fusarium* strain H15. The experiment included 3 types of juvenile plants:

-Uninjured control (Control-Nh),
-Injured control (Control-H), and
-Fungus infected (H15)

Within first month, the holes were re-inoculated twice with the fungus and sterile water was added every 2–3 days into the cotton plugs for another 2 months. Holes in control plants (Control-H) were inoculated with sterile broth instead of the fungus. Tissue from Control-Nh plants were collected before injury. At the end of the experiment, the area of discoloration around the holes was determined using the formula for the area of an ellipse as, π × r1 × r2 (where π = 3.14, r1and r2 = radii of ellipse). Finally, extraction with n-hexane followed by GC-MS analysis was performed to determine the metabolite profiles.

### Fermentation of resinous agarwood chips with associated *Fusarium*

Fermentation of resinous chips was performed by adding the *Fusarium* strain -H15. Firstly, resinous chips were pulverised and subjected to surface sterilisation with 0.1% HgCl2 (w/v) and 70.0% ethanol (v/v) for 1.0 min. each and placed inside sterile 50 ml culture tubes. To 1.0 g wood tissue, 10.0 ml sterile water was added. Fresh culture of *Fusarium* (block scooped by cork borer from PDA plates) was inoculated into the tubes, sealed and placed under ambient conditions for 45 days. The *Fusarium* and resinous wood were subjected to the same steps and incubated under identical conditions to serve as controls. On the 20th day of culture, 50 μl of the liquid from each tube was spread onto fresh PDA plates and incubated at 25 °C to confirm the viability of the fungus by re-isolation. At the end of the experiment, n-hexane extracts of the following metabolic phenotypes were compared by GC-MS:

- *Boha* oil (Bh),
- Un-soaked resinous wood (USR),
- Soaked resinous wood (SR),
- Resinous wood fermented with fungus (RH15), and
- Pure culture of fungus fermented without resinous wood (PH15)

### Solvent extraction and GC-MS analysis

For the samples of diverse nature a simplified approach for metabolite extraction and analysis was adopted. Solvent extraction was preferred (over hydro-distillation) to maximize the chance of detecting diverse compounds. External or internal standards were unnecessary or impossible to use in this untargeted study and instead relative quantities were compared. Therefore, relative abundance (peak area%) with normalization (log transformation) was used as the input data as per guidelines for the quantitative gas chromatography of volatile flavoring substances given by the Working Group on Methods of Analysis of the International Organization of the Flavor Industry^48^. Solvent extraction and GC-MS analysis already optimized for agarwood metabolite profiling in the laboratory was used with modifications^22^. The samples were weighed, homogenised and extracted with 10 volumes of *n*-hexane (Lichrosolv, Merck) for a total of 72 h in a shaker (25 °C, 120 rpm). Resinous and non-resinous wood, agarwood oil were also extracted similarly by dissolving 250 mg wood or oil in 2.5 ml n-hexane. After passing through 0.45 μm syringe filters (MiniSart, Sartorius, Germany), GC- MS analysis was performed on a Shimadzu GC2010 Plus - triple quadrupole (TP-8030) GC-MS/MS system fitted with EB-5MS column (length-30 m, thickness −0.25 μm, ID-0.25 mm). The oven programme started at 70 °C for 2 min and ramped at 20 °C/min up to 140 °C and without holding, again ramped at 5 °C/min to 290 °C and then held for 5 min. A 1.0 μl sample was injected at 280 °C using He as carrier gas (1 ml/min) with a split ratio of 10:1. The mass spectrometer was operated in the electron ionization (EI) mode at 70 eV with an ion source temperature of 200 °C and a continuous scan from 50 to 600 m/z. The peaks were identified by matching the mass spectra with the National Institute of Standards and Technology (NIST, USA) library. Noisy peaks, column bleed (silanes/silxoxanes) were removed from the total ion chromatograms (TIC) before further studies.

### Metabolite profile analysis

Information about individual compounds in the metabolite profile was searched in SciFinder (http://www.cas.org/products/scifinder) as-

- Reported in agarwood (search words: agarwood, *Aquilaria*),
- Reported in aromatics (search words: aroma, essential oil, perfume), and
- Reported in fungus and *Fusarium* (search words: fungus, *Fusarium*).

Reliable databases such as PubChem (http://pubchem.ncbi.nlm.nih.gov.), ChemSpider (http://www.chemspider.com) were also searched.

### Correlation network analysis

To construct the network of co-occurrence of the metabolites obtained from GC-MS analysis, bivariate correlation (Pearson’s correlation) was performed on IBM SPSS Statistics 20 software and the network was generated using Cytoscape 3.3.0 for the compounds showing significant (p < 0.05) positive or negative correlation^49^. Networks were visualized using prefuse force directed layout where the nodes represent the metabolite and the edges represent correlation.

### Statistics

All treatments were replicated thrice and experiments were repeated at least once. Statistical analyses such as principal components analysis (PCA) and analysis of similarity (ANOSIM) were performed using Past 2.7c software. Multiple dataset analysis feature of Vennture^50^ was used to study the distribution of compounds from the GC-MS profiles by Venn analysis. Metaboanalyst^51^ package, customized for metabolomics, was used for fold change analysis, and generation of heat map to compare the change in metabolite levels during interaction. Relative concentration (% peak area) were normalised by log transformation (glog) prior to analysis. Fold change analysis was performed within a threshold maintained at 1.0 and expressed as log2FC.

## Additional Information

**How to cite this article**: Sen, S. *et al*. Chemometric analysis reveals links in the formation of fragrant bio-molecules during agarwood (*Aquilaria malaccensis*) and fungal interactions. *Sci. Rep.* **7**, 44406; doi: 10.1038/srep44406 (2017).

**Publisher's note:** Springer Nature remains neutral with regard to jurisdictional claims in published maps and institutional affiliations.

## Supplementary Material

Supplementary Information
