# Supplementary material for: Chemometric analysis reveals links in the formation of fragrant bio-molecules during agarwood (Aquilaria malaccensis) and fungal interactions
Source: Sci Rep. 2017 Mar 14;7:44406. doi: 10.1038/srep44406 (PMC5349546; doi:10.1038/srep44406)
Supplement: Supplementary Information [file srep44406-s1.doc]

**Supplementary information**

**Title**

Chemometric analysis reveals links in the formation of fragrant bio-molecules during agarwood (*Aquilaria malaccensis*) and fungal interactions

**Authors**

Supriyo Sen^1,2^, Madhusmita Dehingia^1^, Narayan Chandra Talukdar^1^ and Mojibur Khan^1^*

**Affiliation**

^1^Biodiversity & Ecosystem Research Group, Institute of Advanced Study in Science and Technology (IASST), DST, Govt. of India, Guwahati- 781035, Assam, India.

^2^ Present Address: Department of Biotechnology, School of Life Sciences, Assam Don Bosco University, Tapesia, Sonapur, Assam, India

*Corresponding author: mojibur.khan@gmail.com

**Content**

**Supplementary Methods**

**Supplementary Results**

**Supplementary Figures**

**Supplementary Tables**

**Supplementary Methods**

*Isolation of agarwood associated fungi*

To isolate associated fungi, fresh agarwood samples of two distinct types were separately analysed. Dark coloured, resinous wood from fungus infected portions (locally called *Maal-dhara*) and pale brown non-resinous wood from uninfected tissue of *A. malaccensis* were collected separately. The pieces of wood were further cut into smaller pieces and followed by surface sterilisation for 1 min with 70.0 % ethanol. After repeated rinse with sterile water, the wood was vortex mixed (30 min.) to draw the fungal mycelia and spores into the suspension. The contents were then plated onto potato dextrose agar (PDA) media containing streptomycin (50.0 mg/L) and chloramphenicol (50.0 mg/L). After 7-10 days pure cultures of morphologically distinct fungal isolates specific to resinous tissue were selected and maintained separately.

*Callus production from leaves of* A. malaccensis

*In vitro* callus induction from *A. malaccensis* leaf was attempted from tender leaves collected from two year old *A. malaccensis* plants growing in the experimental agarwood plantation of IASST, Guwahati (26°6'36.22"N; 1°41'6.87"E) during the month of June. Surface sterilized leaves (0.1% HgCl_2_ for 3 min and 70.0 % ethanol for 30 s) were cut into pieces and placed abaxially in plates containing modified MS media. To optimize the callus induction medium, commercially available MS basal medium (Himedia, Mumbai) was supplemented with different combinations of BAP (0 - 3.0 µM) and 2, 4 - D (0- 4.0 µM) and pH adjusted to 5.6-5.8. Before being autoclaved at 121°C (15.0 lbs) for 15 mins, plant culture tested agar (Himedia, Mumbai) was added @ 8.0 g/L. Cultures were maintained at 25±2 °C in dark and calli were sub-cultured every 30 days into fresh medium.

*Identification of fungi by ITS sequencing*

Partial ITS region was amplified using the primer pair ITS1 (5'-TCCGTAGGTGAACCTGCGG-3') and ITS4 (5'-TCCTCCGCTTATTGATATGC-3'). PCR reaction was performed in a 25 μL volume in a thermal cycler (Mastercycler Nexus Gradient, Eppendorf, Germany). Each PCR reaction contained a final concentration of 1× standard buffer, 1.5 mM MgCl_2_, 0.2 μM each primer, 0.2 mM dNTPs, and 0.25 U Taq DNA polymerase (Sigma- Aldrich) and 25 ng of template DNA. PCR program consisted of 95 °C for 5 min, followed by 40 cycles of 94 °C for 30 s, 55 °C for 30 s, 72 °C for 45 s, and finally at 72 °C for 7 min. PCR products were separated in a 1.2% agarose gel along with 100 bp DNA ladder and visualized under BioDoc-It Imaging System (UVP). The PCR products were purified using a PCR clean up kit (Sigma-Aldrich). Purified PCR products were sequenced in GenomeLab GeXP capillary Sequencer (Beckman Coulter, USA). The sequences were aligned using BLAST (http://www.ncbi.nlm.nih.gov /BLAST/) tool to identify the isolates.

**Supplementary Results**

*Isolation of agarwood associated fungi*

From resinous wood collected from four different agarwood production centers of Assam, a total of 33 associated fungi were isolated. Only morphologically diverse isolates from resinous wood that had no counterpart in the plates inoculated with non-resinous wood were assigned individual codes. The recovery of isolates varied across locations. Sample from Hojai with 12 fungi was the source of the highest number of isolates closely followed by Janji (11), while Nahorani (6) and Namti (4) contributed the rest (Supplementary Table ST-1).

*Callus from leaves of A. malaccensis*

Initiation of callus was indicated by curling and malformation of the responding explants, 6-7 days after inoculation. Callus induction frequency and rate of proliferation of callus was found to be highest in MS media supplemented with 3.0 µM of each of 2,4-D. In this media calli were formed in 83.25% explants, appeared creamy in colour and were moderately friable to touch (Supplementary Table ST-2). Callus proliferation was uniform over a period of 60 days with a 30 day interval of sub-culture. From the same culture, clumps of callus that were dry, friable with uniform appearance were multiplied in bulk for use in the subsequent experiments.

*Interaction of A. malaccensis callus with associated Fusarium isolate (H15)*

In a preliminary experiment, *Fusarium* (H15) mycelia were found to interact and physically cover the callus co-cultured in a Petri plate (Supplementary Figure SF-2a, b). Evan’s test showed the callus remained viable till at least a month of association (Supplementary Figure SF-2c). Rate of growth of fungal biomass was evidently faster compared to callus, but was restrained during interaction.

*Compounds shared by callus and fungus*

A fewer number of compounds were expected to be shared by callus and fungus. A total of 6 such compounds were identified in the profiles of callus and fungus (Supplementary Table ST- 7). Out of these an alkane (dotriacontane) is reported in profile of agarwood.

*Compounds present only in callus*

Totally 42 compounds were detected in the profile of callus that were absent in fungus as well as interaction. They were mostly alkanes/aklenes (16) and esters (13) while aldehydes/ ketones (5), alcohols (2) and others (6) comprised the rest of the profile (Supplementary Table ST- 8). 18 out of the 42 compounds are mentioned in the aromatic profiles of essential oils in literature out of which 5 are reported in agarwood profiles.

*Compounds present only in fungus*

A total of 51 compounds were unique to *Fusarium* (Supplementary Table ST-9). The profile comprised of esters (16), alkanes/alkenes (15), alcohols (7), aldehydes/ ketones (5) and others (8). 16 of the compounds are reported in profiles of fungi as well as essential oils and out of them 7 compounds are reported in agarwood indicating aroma compound production by fungi when grown alone.

**Supplementary Figures**


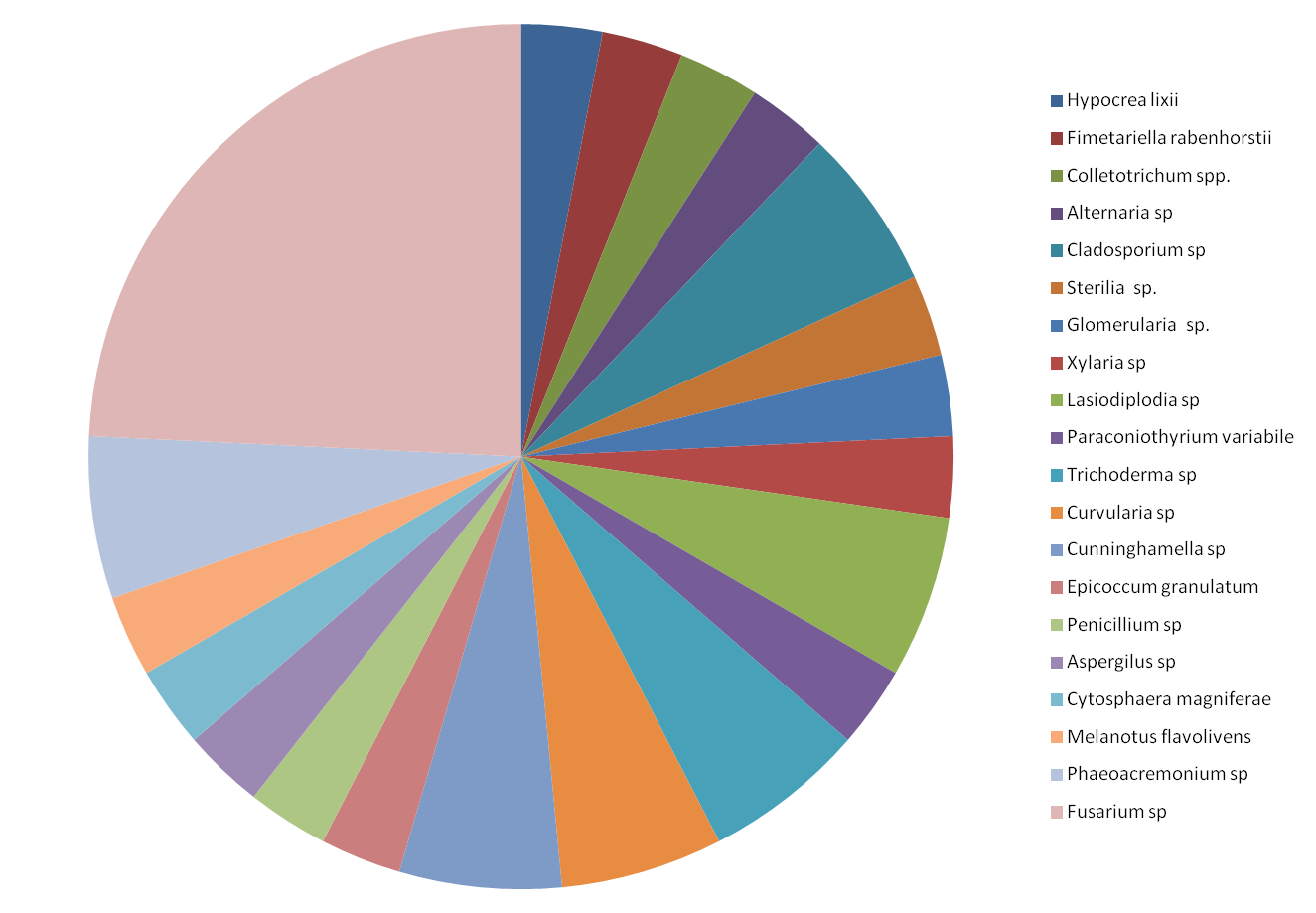


**Supplementary Figure SF-1:** Pie diagram representing fungal genera associated with agarwood tissue, based on data available from published literature on agarwood.


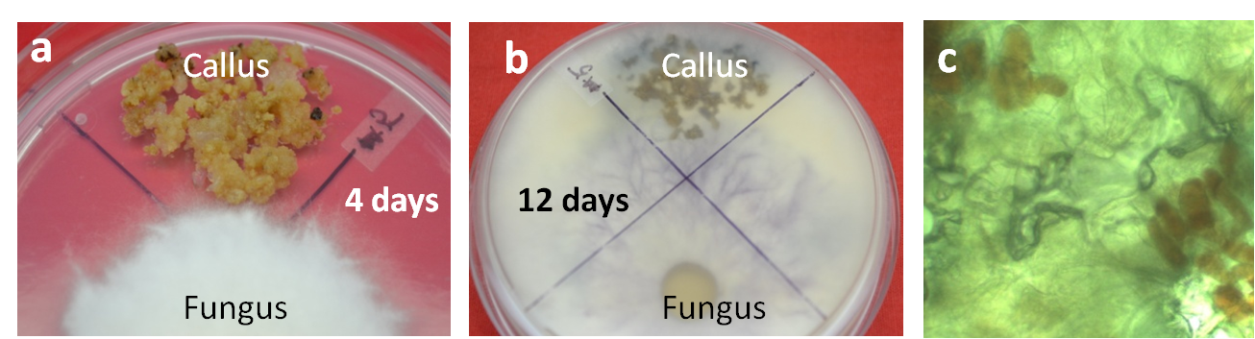


**Supplementary Figure SF-2: Co-culture of *Aquilaria* callus and *Fusarium*.** (a) Mycelia approaching the callus tissue (4 days after co-culture), (b) Mycelia covered the entire plate along with the callus after 12 days of co-culture, (c) Evan’s test after 30 days of co-culture.


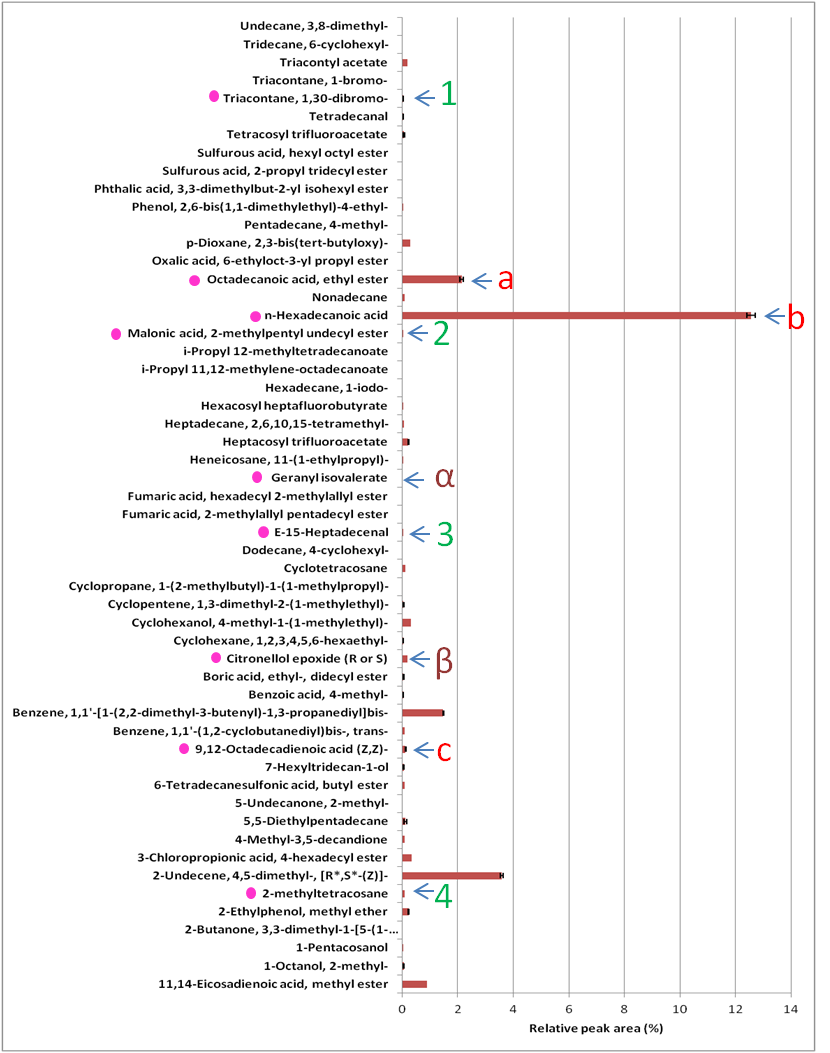


**Supplementary Figure SF-3: Compounds formed only during interaction:** Relative peak area (%) of the 54 compounds unique to the profile of interaction Note their relation to fatty acid metabolism (a,b,c), wax biosynthesis (1,2,3,4) and terpenoids ( α, β).


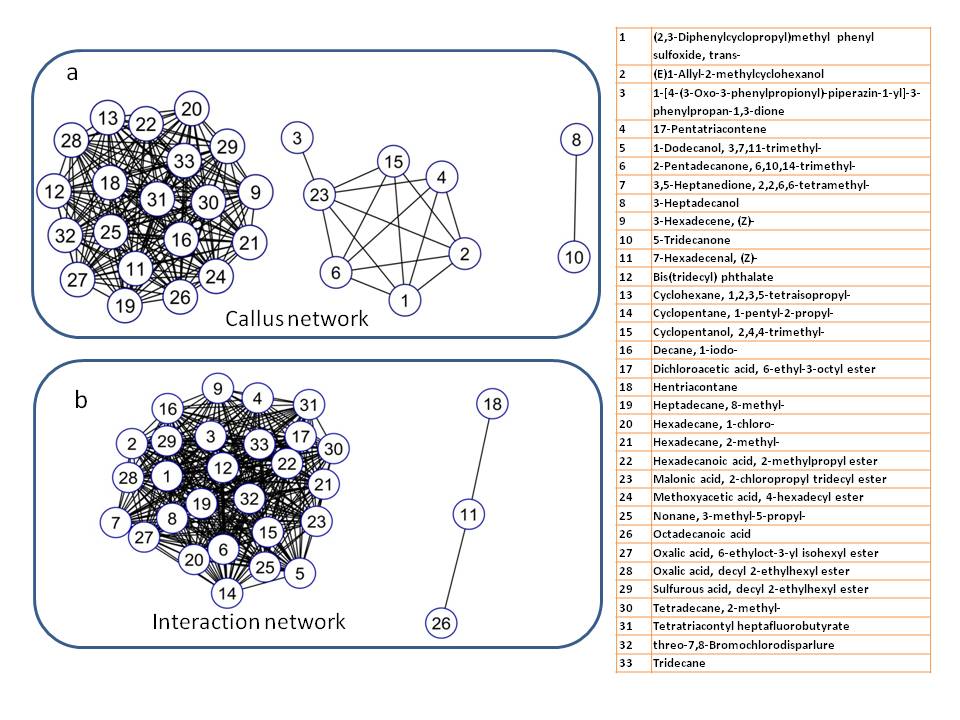


**Supplementary Figure SF-4: Correlation network of compounds shared by callus and interaction (i.e. not fungus)**. Network analysis for the compounds based on significant correlation existing in case of, a. Callus (29 out of 33 compounds) and b. Interaction (30 out of 33 compounds).


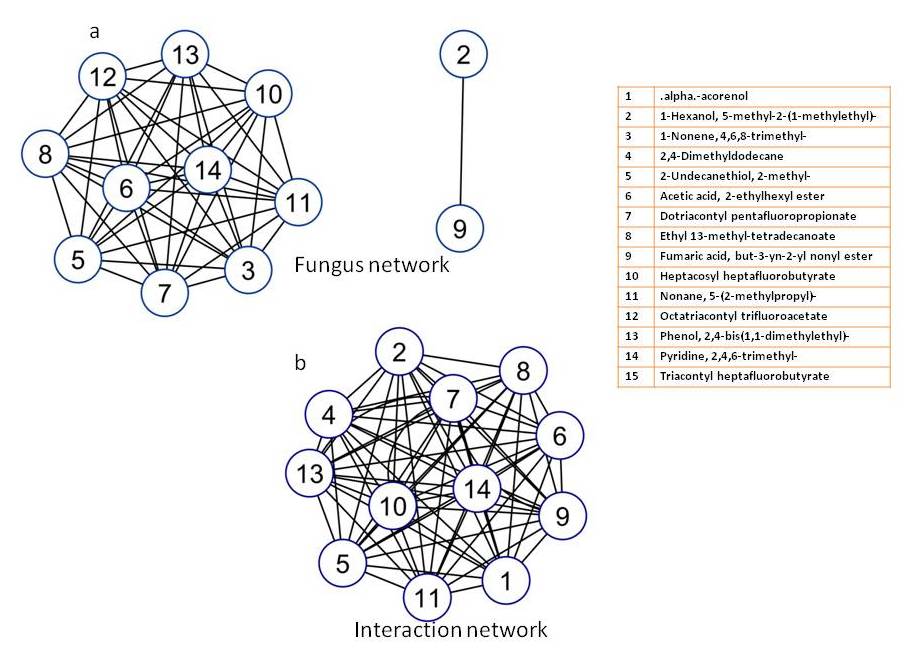


**Supplementary Figure SF-5: Correlation network of compounds shared by fungus and interaction (i.e. not callus)**. Network analysis for the compounds based on significant correlation (12 out of 15 compounds) in case of, a. Fungus and b. Interaction.

**Supplementary tables**

**Supplementary Table ST 1: Fungal isolates from infected samples of agarwood from different geographical sites of Assam**

| **Sl. No.** | **Site** | **Reference code^1^** | **Short code^2^** | **Morphological description^3^** |
| --- | --- | --- | --- | --- |
| 1 | **Janji** | Old (1)36 | J3 | Yellow to white cottony mass |
| 2 |  | Old (4) 39 | J4 | Dirty white cottony mass |
| 3 |  | S2 (1) 16 | J5 | Black cottony mass with radiating streaks |
| 4 |  | P2 (4) 14 | J6 | Reddish brown colonies |
| 5 |  | Old (3) 38 | J7 | Dark brown to black colonies; slow growing |
| 6 |  | P2(2) 12 | J8 | Whitish colonies with red centre and pink margin |
| 7 |  | P2 (5) 15 | J9 | Light yellow to white cottony mass |
| 8 |  | S2(2) 17 | J10 | Brown slow growing colony |
| 9 |  | P2 (1) 11 | J11 | Dirty brown mass with white border |
| 10 |  | Old (2) 37 | J12 | Yellow colonies with brownish centre |
| 11 |  | P2 (3) 13 | J13 | Greyish brown cottony fast growing mass |
| 12 | **Hojai** | P4 (4) 33 | H14 | Cottony mass with green centre and lighter margin |
| 13 |  | S4 (4) 25 | H15 | Cottony mass of white gradually turning pinkish |
| 14 |  | P4 (1) 30 | H16 | Light green mass with whitish margin |
| 15 |  | S4 (5) 26 | H17 | Mass with green centre and narrow white margin |
| 16 |  | S4 (6.1) 27 | H18 | Dark black slimy slow growing colony |
| 17 |  | S4 (1) 22 | H19 | Colony with reddish centre and white margin |
| 18 |  | S4 (6.2) 28 | H20 | Dirty green colonies |
| 19 |  | S4 (2) 23 | H21 | Dark green cottony mass (appears black) |
| 20 |  | S4 (3) 24 | H22 | Dark green cottony mass with slimy black centre |
| 21 |  | S4 (6.3) 29 | H23 | Cottony mass with pale yellow appearance |
| 22 |  | P4 (2) 31 | H24 | Green mass with dark centre and reddish margin |
| 23 |  | P4 (3) 32 | H25 | Brown cottony mass |
| 24 | **Namti** | S8 (1) 4 | NM26 | Whitish mass with streaks radiating from centre |
| 25 |  | S8 (2) 3 | NM27 | Black colony |
| 26 |  | P8 (3) 2 | NM28 | White colony |
| 27 |  | P8 (2) 1 | NM29 | White leathery colony |
| 28 | **Nahorani** | S6 (1) 5 | NH30 | Green mass with white border |
| 29 |  | S6 (2) 6 | NH31 | Green mass with narrow white border |
| 30 |  | S6 (4) 8 | NH32 | Colony with greenish centre and white mycelia |
| 31 |  | P6 (2) 10 | NH33 | Light green cottony fast growing mass |
| 32 |  | P6 (1) 9 | NH34 | Dark green cottony fast growing mass |
| 33 |  | S6 (3) 7 | NH35 | Black powdery colonies |

^1^ Reference and ^2^ Short codes were assigned to each isolate to refer to the experimental design and site of sample collection, respectively. **^3^** Surface characters viewed from top of PDA plate after 7-10 days of inoculation.

**Supplementary Table ST2:** Callus induction from tender leaf explants of *A. malaccensis*

| **Growth regulators (µM)** | | **Callus induction** (30 days of culture) | |
| --- | --- | --- | --- |
| **2,4-D** | **BAP** | **Frequency (%)*** | **Morphology** |
| 0 | 0 | 6.75^f^ | White; not well formed |
| 1.5 | 0 | 15.30^ef^ | Translucent; slow growing |
| 3.0 | 0 | 28.78^de^ | Translucent; slow growing |
| 4.0 | 0 | 41.15^cd^ | Cream colour; compact |
| 3.0 | 1.5 | 56.99^b^ | Cream colour; compact |
| 3.0 | 3.0 | 83.25^a^ | Cream colour; moderately friable |
| 4.0 | 1.5 | 54.78^bc^ | White ; compact to friable |
| 4.0 | 3.0 | 63.93^b^ | Translucent; compact to friable |
|  |  | CV = 18.19; CD(0.05)=13.82 |  |

*ANOVA carried out for CRD in WASP - Web Agri Stat Package; (http:// icargoa.res.in/ wasp /index.php).

**Supplementary Table ST-3: Compounds unique to interaction**

| **Chemical class** | **Name of the compound** | **Chemical identity** | **Reported in essential oil/perfume^1^** | **Reported in agarwood^1^** | **Additional information^2^** |
| --- | --- | --- | --- | --- | --- |
| Acid esters | Boric acid, ethyl-, didecyl ester | CAS 332952-33-7 | N | N |  |
|  | Malonic acid, 2-methylpentyl undecyl ester | InChI Key: YMIDJZBIDXUYAM-UHFFFAOYSA-N | N | N | Malonyl-CoA is an intermediate in the synthesis of fatty acids in plants. |
|  | Sulfurous acid, 2-propyl tridecyl ester | PubChem CID: 64 20355 | Y | N |  |
|  | Oxalic acid, 6-ethyloct-3-yl propyl ester | ChemSpider ID 4925969 | Y | N |  |
|  | Phthalic acid, 3,3-dimethylbut-2-yl isohexyl ester | InChIKey: ASXIHOZXNTWIPE-UHFFFAOYSA-N | Y | N |  |
|  | Fumaric acid, 2-methylallyl pentadecyl ester | Information unavailable | NA | NA | Fumaric acid production by submerged fermentation. |
|  | Sulfurous acid, hexyl octyl ester | CAS 959067-59-5 | Y | N |  |
|  | Fumaric acid, hexadecyl 2-methylallyl ester | Information unavailable | NA | NA | Fumaric acid production by submerged fermentation. |
| Acids | Hexacosyl heptafluorobutyrate | InChI Key  QHZFIZAXWYXCPT-UHFFFAOYSA-N | N | N |  |
|  | Benzoic acid, 4-methyl- | CAS 99-94-5 | Y | N |  |
|  | i-Propyl 12-methyltetradecanoate | CAS 110-20-0 | Y | Y | Reported in agarwood fermentation as well as *Fusarium*; used in absorbing and dilution of perfumes; impart ability to retain aroma. |
|  | i-Propyl 11,12-methylene-octadecanoate | Information unavailable | NA | NA |  |
| Alcohol acetates | Heptacosyl trifluoroacetate | ChemSpider ID-29739974 | NA | NA |  |
|  | Triacontyl acetate | CAS - 41755-58-2 | Y | N |  |
|  | Tetracosyl trifluoroacetate | InChIKey: DOXMLSKFEKEMHX-UHFFFAOYSA-N | NA | NA |  |
| Alcohols | Cyclohexanol, 4-methyl-1-(1-methylethyl)- | CAS - 470-65-5 | Y | N | Menthol like volatile also produced by fungi (eg. *Muscador* spp ). |
|  | 1-Octanol, 2-methyl- | CAS 818-81-5 | Y | N |  |
|  | 1-Pentacosanol | CAS 26040-98-2 | Y | N |  |
|  | 7-Hexyltridecan-1-ol | CAS 959046-51-6 | Y | N |  |
| Aldehydes | E-15-Heptadecenal | CAS 988581-82-2 | Y | N |  |
|  | Tetradecanal | CAS 124-25-4 | Y | Y | Antifungal effect on *Fusarium* and other fungi. Constituent of fungal aroma. |
| Alkanes | Cyclotetracosane | CAS 297-03-0 | Y | Y |  |
|  | 2-methyltetracosane | CAS 1560-78-7 | Y | Y |  |
|  | Nonadecane | CAS 629-92-5 | Y | Y | Important perfume component |
|  | Heptadecane, 2,6,10,15-tetramethyl- | CAS 54833-48-6 | Y | Y |  |
|  | Heneicosane, 11-(1-ethylpropyl)- | CAS 55-282-11-6 | Y | N |  |
|  | Triacontane, 1,30-dibromo- | CAS 121473-35-6 | Y | N |  |
|  | 5,5-Diethylpentadecane | InChIKey: LUCOSBOKHVFCQX-UHFFFAOYSA-N | N | N |  |
|  | Triacontane, 1-bromo- | CAS 4209-22-7 | Y | N |  |
|  | Dodecane, 4-cyclohexyl- | CAS 13151-84-3 | Y | N |  |
|  | Cyclohexane, 1,2,3,4,5,6-hexaethyl- | CAS 1795-14-8 | N | N |  |
|  | Cyclopropane, 1-(2-methylbutyl)-1-(1-methylpropyl)- | CAS 64723-36-0 | Y | N |  |
|  | Undecane, 3,8-dimethyl- | CAS 17301-30-3 | Y | N |  |
|  | Pentadecane, 4-methyl- | CAS 2801-87- | Y | N | Fungal volatile |
|  | Tridecane, 6-cyclohexyl- | CAS 13151-91-2 | N | N |  |
|  | Hexadecane, 1-iodo- | CAS 544-77-4 | Y | Y |  |
| Alkenes | 2-Undecene, 4,5-dimethyl-, [R*,S*-(Z)]- | PubChem ID:5364969 | NA | NA |  |
|  | Cyclopentene, 1,3-dimethyl-2-(1-methylethyl)- | CAS 61142-32-3 | N | N |  |
| Aromatics | Benzene, 1,1'-[1-(2,2-dimethyl-3-butenyl)-1,3-propanediyl]bis- | CAS - 61142-62-9 | Y | N |  |
|  | Benzene, 1,1'-(1,2-cyclobutanediyl) bis-, trans- | CAS 20071-09-4 | Y | N |  |
|  | 2-Ethylphenol, methyl ether | CAS - 10568-38-4 | Y | N |  |
| Ethers | p-Dioxane, 2,3-bis(tert-butyloxy)- | PubChem [CID:545346](cid:545346) | NA | NA |  |
| Fatty acid esters | 11,14-Eicosadienoic acid, methyl ester | CAS 2463-02-7 | Y | N |  |
|  | 3-Chloropropionic acid, 4-hexadecyl ester | CAS 53312-70-2 | N | N |  |
|  | Octadecanoic acid, ethyl ester  (Stearic acid ester) | CAS -111-61-5 | Y | Y | Carrier for fragrant molecules |
|  | 6-Tetradecanesulfonic acid, butyl ester | CAS 958990-87-9 | Y | N |  |
| Fatty acids | n-Hexadecanoic acid (Palmitic acid) | CAS -57-10-3 | Y | Y |  |
|  | 9,12-Octadecadienoic acid (Z,Z)-  (Linoleic acid) | CAS 60-33-3 | Y | Y | Lipoxygenase pathway or Octadecanoate pathway leading to Jasmonate production. |
| Ketones | 4-Methyl-3,5-decandione | Information unavailable | NA | NA |  |
|  | 5-Undecanone, 2-methyl- | CAS 50639-02-6 | Y | N |  |
|  | 2-Butanone, 3,3-dimethyl-1- | Information unavailable | NA | NA | 2-butanone is a fragrant molecule |
| Phenols | Phenol, 2,6-bis(1,1-dimethylethyl)-4-ethyl- | CAS 1795-14-8 | N | N |  |
| Terpenoids | Citronellol epoxide (R or S) | CAS -1564-98-3 | Y | N |  |
|  | Geranyl isovalerate | CAS 109-20-6 | Y | Y | Semiochemical/Pheromone;  Also in *Fusarium* spp. |

^1^Search results of SciFinder (Y= yes; N= No; NA = Information not available in SciFinder). ^2^ Information from available literature, database, etc.

**Supplementary Table ST-4:** Compounds shared by callus and interaction (i.e. not fungus)

**Supplementary Table -ST-4.1:** Table showing fold changes between compounds shared by callus and interaction (i.e. not fungus)

| **Sl. No.** | **Compound** | **Fold Change (interaction/ callus)** | **log2(FC) (interaction/ callus)** | **Remark** |
| --- | --- | --- | --- | --- |
| 1 | Bis(tridecyl) phthalate | 56.375 | 5.817 | Compounds that showed major increase during interaction (fold change log2FC >1.0) |
| 2 | 17-Pentatriacontene | 7.7544 | 2.955 |
| 3 | Cyclohexane, 1,2,3,5-tetraisopropyl- | 7.3494 | 2.8776 |
| 4 | Methoxyacetic acid, 4-hexadecyl ester | 5.929 | 2.5678 |
| 5 | threo-7,8-Bromochlorodisparlure | 4.4546 | 2.1553 |
| 6 | Tetratriacontyl heptafluorobutyrate | 4.3239 | 2.1123 |
| 7 | (2,3-Diphenylcyclopropyl)methyl phenyl sulfoxide, trans- | 4.2302 | 2.0807 |
| 8 | 5-Tridecanone | 3.4493 | 1.7863 |
| 9 | Sulfurous acid, decyl 2-ethylhexyl ester | 3.1746 | 1.6666 |
| 10 | 3-Heptadecanol | 2.8792 | 1.5256 |
| 11 | 3-Hexadecene, (Z)- | 2.6799 | 1.4222 |
| 12 | 1-[4-(3-Oxo-3-phenylpropionyl)-piperazin-1-yl]-3-phenylpropan-1,3-dione | 2.3002 | 1.2018 |
| 13 | Tetradecane, 2-methyl- | 2.1508 | 1.1049 |
| 14 | Nonane, 3-methyl-5-propyl- | 1.9509 | 0.96413 | Compounds that showed moderate to low increase during interaction (fold change; log2FC = 0-1.0) |
| 15 | 3,5-Heptanedione, 2,2,6,6-tetramethyl- | 1.9357 | 0.95283 |
| 16 | Tridecane | 1.9097 | 0.93332 |
| 17 | Hexadecanoic acid, 2-methylpropyl ester | 1.6863 | 0.75382 |
| 18 | Dichloroacetic acid, 6-ethyl-3-octyl ester | 1.6501 | 0.72256 |
| 19 | Cyclopentanol, 2,4,4-trimethyl- | 1.6349 | 0.70918 |
| 20 | 2-Pentadecanone, 6,10,14-trimethyl- | 1.4554 | 0.54137 |
| 21 | Oxalic acid, 6-ethyloct-3-yl isohexyl ester | 1.3734 | 0.45773 |
| 22 | Hexadecane, 1-chloro- | 1.2721 | 0.3472 |
| 23 | Oxalic acid, decyl 2-ethylhexyl ester | 1.2404 | 0.31084 |
| 24 | Malonic acid, 2-chloropropyl tridecyl ester | 1.1154 | 0.15754 |
| 25 | Decane, 1-iodo- | 1.0665 | 0.09286 |
| 26 | Cyclopentane, 1-pentyl-2-propyl- | 1.0408 | 0.05769 |
| 27 | 7-Hexadecenal, (Z)- | 1.0239 | 0.03404 |
| 28 | Hentriacontane | 0.82822 | -0.27191 | Compounds that decreased during interaction (log2FC<0) |
| 29 | 1-Dodecanol, 3,7,11-trimethyl- | 0.77729 | -0.36347 |
| 30 | Octadecanoic acid | 0.6094 | -0.71454 |
| 31 | Heptadecane, 8-methyl- | 0.44188 | -1.1783 |
| 32 | Hexadecane, 2-methyl- | 0.28534 | -1.8093 |
| 33 | (E)1-Allyl-2-methylcyclohexanol | 0.12396 | -3.0121 |

**Supplementary Table -ST- 4.2: Compounds high in callus and decreased during interaction**

| **Chemical class** | **Name of the compound** | **Chemical identity** | **Reported in essential oil/perfume^1^** | **Reported in agarwood^1^** | **Additional information^2^** |
| --- | --- | --- | --- | --- | --- |
| Alcohols | (E)1-Allyl-2-methylcyclohexanol | CAS 24580-51-6 | N | N |  |
|  | 1-Dodecanol, 3,7,11-trimethyl-  (Farnesol) | CAS 6750-34-1 | Y | Y | Terpene alcohol; fragrant |
| Alkanes | Hexadecane, 2-methyl- | CAS 1560-92-5 | Y | N | Component of plant epicuticular wax. Present in fungi. Also reported in control of *Fusarium proliferatum* by volatiles. |
|  | Heptadecane, 8-methyl- | CAS 13287-23-5 | Y | N | Epicuticular wax component; Reported from fungus. |
|  | Hentriacontane | CAS Registry Number 630-04-6 | Y | Y | Major component of cuticular waxes |
| Fatty acid | Octadecanoic acid  (Stearic acid) | CAS 57-11-4 | Y | Y | The apoplastic oxidative burst in response to biotic stress in plants. Possibly converted into ethyl stearate. |

^1^Search results of SciFinder (Y= yes; N= No; NA = Information not available in SciFinder). ^2^ Information from available literature, database, etc.

**Supplementary Table -ST- 4.3.1:** Compounds low in callus and increased during interaction [major fold change **(**log2 fold change>1.0)]

| **Chemical class** | **Name of the compound** | **Chemical identity** | **Reported in essential oil/perfume^1^** | **Reported in agarwood^1^** | **Additional information^2^** |
| --- | --- | --- | --- | --- | --- |
| Acid esters | Bis(tridecyl) phthalate | CAS 119-06-2 | N | N | Perfume component; solvent in perfume |
|  | Methoxyacetic acid, 4-hexadecyl ester | ChemSpider ID 475214 | Y | N |  |
|  | Sulfurous acid, decyl 2-ethylhexyl ester | CAS 959311-40-1 | N | N | Role in plant - fungus interaction |
| Acids | Tetratriacontyl heptafluorobutyrate | InChIKey: DGGUHEYSIUTPKS-UHFFFAOYSA-N | N | N |  |
| Alcohols | 3-Heptadecanol | CAS 84534-30-5 | Y | N | Aroma profiles, essential oils; Perfume fixative. |
| Alkanes | Cyclohexane, 1,2,3,5-tetraisopropyl- | CAS 854449-17-5 | N | N |  |
|  | Threo-7,8-Bromochlorodisparlure | ChemSpider ID 468213 | N | N | Disparlure is a synthetic sex pheromone for insects. |
|  | Tetradecane, 2-methyl- | CAS 1560-95-8 | Y | Y | Reported artificial induction by pinhole infusion techniques in agarwood. |
| Alkenes | 17-Pentatriacontene | CAS 6971-40-0 | Y | Y | Component of perfumes, essential oils |
|  | 3-Hexadecene, (Z)- | CAS 34303-81-6 | Y | N | Aroma profiles, essential oils |
| Ketones | 5-Tridecanone | CAS 30692-16-1 | Y | N |  |
|  | 1-[4-(3-Oxo-3-phenylpropionyl)-piperazin-1-yl]-3-phenylpropan-1,3-dione | PubChem CID 237317 | N | N |  |
| Sulfoxides | (2,3-Diphenylcyclopropyl)methyl phenyl sulfoxide, trans- | CHEBI ID 84273 | Y | N | Essential oils; many sulfoxides are fragrant compounds |

^1^Search results of SciFinder (Y= yes; N= No; NA = Information not available in SciFinder). ^2^ Information from available literature, database, etc.

**Supplementary Table- ST- 4.3.2:** Compounds low in callus and increased during interaction [medium to no fold change (log2 fold change 0.0-1.0)]

| **Chemical class** | **Name of the compound** | **Chemical identity** | **Reported in essential oil/perfume^1^** | **Reported in agarwood^1^** | **Additional information^2^** |
| --- | --- | --- | --- | --- | --- |
| Alkanes | Nonane, 3-methyl-5-propyl- | CAS 31081-18-2 | Y | N |  |
| Acid esters | Dichloroacetic acid, 6-ethyl-3-octyl ester | PubChem CID 550157 | NA | NA |  |
|  | Oxalic acid, 6-ethyloct-3-yl isohexyl ester | PubChem CID 6420419 | NA | NA |  |
|  | Oxalic acid, decyl 2-ethylhexyl ester | ChemSpider ID 4926337 | NA | NA |  |
|  | Malonic acid, 2-chloropropyl tridecyl ester | InChIKey: XQULABOIZJGPFB-UHFFFAOYSA-N | N | N | Malonyl-CoA is an intermediate in the synthesis of fatty acids and some aromatic compounds of plants and moulds. |
| Alcohols | Cyclopentanol, 2,4,4-trimethyl- | CAS 56470-83-8 | Y | N | Fragrant compound |
| Aldehydes | 7-Hexadecenal, (Z)- | CAS 56797-40-1 | Y | N | Sex pheromone |
| Alkanes | Tridecane | CAS 629-50-5 | Y | Y | Role in defense (jasmonate, salicylate pathways) |
|  | Hexadecane, 1-chloro- | CAS 4860-03-1 | Y | N |  |
|  | Decane, 1-iodo- | CAS 2050-77-3 | Y | N | Use as surfactant |
|  | Cyclopentane, 1-pentyl-2-propyl- | CAS 62199-51-3 | Y | N |  |
| Fatty acid esters | Hexadecanoic acid, 2-methylpropyl ester  (Palmitic acid isobutyl ester) | CAS 110-34-9 | Y | N | Free fatty acid synthesis, oxidative burst, defense response. |
| Ketones | 3,5-Heptanedione, 2,2,6,6-tetramethyl- | CAS 1118-71-4 | Y | N |  |
|  | 2-Pentadecanone, 6,10,14-trimethyl-  (Hexahydrofarnesyl acetone) | CAS 502-69-2 | Y | N | Farnesyl acetones are fragrant compounds; substrate |

^1^Search results of SciFinder (Y= yes; N= No; NA = Information not available in SciFinder). ^2^ Information from available literature, database, etc.

**Supplementary Table ST-5:** Compounds shared by fungus and interaction (i.e. not callus)

**Supplementary Table - ST-5.1: Table showing fold changes between compounds shared in the profiles of fungus and interaction (i.e. not callus)**

| **Sl. No.** | **Compound** | **Fold Change (interaction/ fungus)** | **log2(FC) (interaction/ fungus)** | **Remark** |
| --- | --- | --- | --- | --- |
| 1 | Triacontyl heptafluorobutyrate | 2.3887 | 1.2562 | Compounds that increased during interaction (log2FC> 0) |
| 2 | Octatriacontyl trifluoroacetate | 1.5412 | 0.62403 |
| 3 | 2-Undecanethiol, 2-methyl- | 1.37 | 0.4542 |
| 4 | Phenol, 2,4-bis(1,1-dimethylethyl)- | 1.0531 | 0.074699 |
| 5 | 2,4-Dimethyldodecane | 0.78191 | -0.35492 | Compounds that decreased during interaction (log2FC< 0) |
| 6 | Ethyl 13-methyl-tetradecanoate | 0.52382 | -0.93286 |
| 7 | Heptacosyl heptafluorobutyrate | 0.44566 | -1.166 |
| 8 | .alpha.-acorenol | 0.42885 | -1.2215 |
| 9 | Pyridine, 2,4,6-trimethyl- | 0.42142 | -1.2467 |
| 10 | Nonane, 5-(2-methylpropyl)- | 0.39135 | -1.3535 |
| 11 | 1-Hexanol, 5-methyl-2-(1-methylethyl)- | 0.37504 | -1.4149 |
| 12 | Acetic acid, 2-ethylhexyl ester | 0.29046 | -1.7836 |
| 13 | 1-Nonene, 4,6,8-trimethyl- | 0.25313 | -1.9821 |
| 14 | Dotriacontyl pentafluoropropionate | 0.070989 | -3.8163 |  |
| 15 | Fumaric acid, but-3-yn-2-yl nonyl ester | 0.052985 | -4.2383 |

**Supplementary Table -ST-5.2.: Compounds high in fungus and decreased during interaction**

| **Chemical class** | **Name of the compound** | **Chemical identity** | **Reported in essential oil/perfume^1^** | **Reported in agarwood^1^** | **Additional information^2^** |
| --- | --- | --- | --- | --- | --- |
| Acid esters | Fumaric acid, but-3-yn-2-yl nonyl ester | Information unavailable | NA | NA |  |
|  | Acetic acid, 2-ethylhexyl ester | CAS 103-09-3 | E, F | N | Component of several fragrant profiles including *Fusarium* |
| Acids | Dotriacontyl pentafluoropropionate | ChemSpider ID 29740114 | NA | NA |  |
|  | Heptacosyl heptafluorobutyrate | InChIKey: FWXWZVBDHSMBHR-UHFFFAOYSA-N | N | N |  |
|  | Ethyl 13-methyl-tetradecanoate | ChemSpider ID29757472 | E | N |  |
| Alcohols | 1-Hexanol, 5-methyl-2-(1-methylethyl)- | CAS 2051-33-4 | E, F | N |  |
| Alkanes | Nonane, 5-(2-methylpropyl)- | CAS 62185-53-9 | E | N |  |
|  | 2,4-Dimethyldodecane | CAS 6117-99-3 | E | N |  |
| Alkenes | 1-Nonene, 4,6,8-trimethyl- | CAS 54410-98-9 | E | N |  |
| Heterocyclic organic compounds | Pyridine, 2,4,6-trimethyl- | CAS 108-75-8 | E, F | N | Component of several fragrant profiles including *Fusarium* |
| Spiro compound | .alpha.-acorenol  [Spiro[4.5]​dec-​7-​ene-​1-​methanol, α,​α,​4,​8-​tetramethyl-​, (1R,​4R,​5S)​-] | CAS 28296-85-7 | E,F | N | Component of several fragrant profiles as well as *Fusarium.;* Spiro compound; fragrant |

^1^Search results of SciFinder (E= essential oil/aroma/perfume; F= Fungus including *Fusarium*; Y = Yes; N= No; NA = Information not available in SciFinder). ^2^ Information from available literature, database, etc.

**Supplementary Table - ST-5.3.:** Compounds low in fungus and increased during interaction

| **Chemical class** | **Name of the compound** | **Chemical identity** | **Reported in essential oil/perfume^1^** | **Reported in agarwood^1^** | **Additional information^2^** |
| --- | --- | --- | --- | --- | --- |
| Acids | Triacontyl heptafluorobutyrate | - ChemSpider ID 29740103 | N | N |  |
| Alcohol acetates | Octatriacontyl trifluoroacetate | ChemSpider ID29740068 | N | N |  |
| Phenols | Phenol, 2,4-bis(1,1-dimethylethyl)- | CAS 96-76-4 | E, F | Y | Component of several fragrant profiles including *Fusarium* ; Important component of agarwood. |
| Thiols | 2-Undecanethiol, 2-methyl- | CAS 10059-13-9 | E | N |  |

^1^Search results of SciFinder (E= essential oil/aroma/perfume; F= Fungus including *Fusarium*; Y = Yes; N= No; NA = Information not available in SciFinder). ^2^ Information from available literature, database, etc.

**Supplementary Table ST-6:** Compounds shared by all three (fungus, callus and interaction)

| **Chemical class** | **Name of the compound** | **Chemical identity** | **Reported in essential oil/fungus^1^** | **Reported in agarwood^1^** | **Additional information^2^** | **Fold change during interaction (log2FC)** |
| --- | --- | --- | --- | --- | --- | --- |
| Acid esters | Phthalic acid, diisobutyl ester | CAS 84-69-5 | E,F | Y | Important component of perfumes. | Int./Callus: (-) 0.01  Int/Fungus: 0.076 |
|  | Isodecyl methacrylate | CAS 29964-84-9 | E | N |  | Int./Callus: 1.326  Int/Fungus: (-)0.547 |
|  | Bis(2-ethylhexyl) phthalate | CAS 117-81-7 | E, F | Y | Important component of perfumes. | Int./Callus: 0.483  Int/Fungus:1.602 |
|  | Octatriacontyl pentafluoropropionate | CAS 1499194-09-0 | E | N |  | Int./Callus: 2.545  Int/Fungus:0.959 |
|  | Oxalic acid, bis(6-ethyloct-3-yl) ester | PubChem CID 6420814 | N | N |  | Int./Callus: 0.906 Int/Fungus: 1.025 |
|  | Pentanoic acid, 2,2,4-trimethyl-3-carboxyisopropyl, isobutyl ester | PubChem CID 551220 | E, F | N | Aroma compound | Int./Callus: 0.248 Int/Fungus: 2.6484  (2^nd^ highest increase) |
|  | Hexanedioic acid, bis(2-ethylhexyl) ester  (Adipic acid, bis(2-ethylhexyl) ester) | CAS 103-23-1 | E, F | N | Adipic acid derivatives are known as elicitors of defense response. | Int./Callus: (-) 6.709  **(**Highest decrease)  Int/Fungus: 0.468 |
|  | Isopropyl myristate  (Tetradecanoic acid, 1-​methylethyl ester) | CAS 110-27-0 | E, F | Y | Fragrant, agarwood | Int./Callus: 0.601  Int/Fungus: (-) 0.328 |
| Alcohol acetates | Hexatriacontyl trifluoroacetate | ChemSpider ID 29740067 | N | N |  | Int./Callus: 0.187  Int/Fungus: 0.337 |
| Alcohols | 11-Methyldodecanol | CAS 85763-57-1 | N | N | Volatile formed during fermentation of fats and oils. | Int./Callus: 0.630  Int/Fungus:0.540 |
|  | [1,1'-Biphenyl]-2,3'-diol, 3,4',5,6'-tetrakis(1,1-dimethylethyl)- | ChemSpider ID 29740223 | N | N |  | Int./Callus: (-) 0.026  Int/Fungus: 0.865 |
|  | 1-Nonanol, 4,8-dimethyl- | CAS 33933-80-1 | E | Y | Agarwood , fragrant | Int./Callus: 1.625 Int/Fungus: (-)1.286 |
|  | 1-Octanol, 2-butyl- | CAS 3913-02-8 | E, F | N | Constituent of aroma profiles | Int./Callus: 2.668  Int/Fungus: 0.490 |
|  | 1-Hexanol, 2-ethyl- | CAS 104-76-7 | E, F | Y | *Fusarium*; fragrant | Int./Callus: 3.328 (High increase )  Int/Fungus: (-) 0.575 |
|  | 2-Isopropyl-5-methyl-1-heptanol | CAS 91337-07-4 | E | N |  | Int./Callus: 1.32 Int/Fungus: (-) 2.058 |
|  | 1-Decanol, 2-hexyl- | CAS 2425-77-6 | E | N |  | Int./Callus: (-) 4.567 Int/Fungus: (-) 2.783 |
| Aldehydes | Benzaldehyde, 4-methyl- | CAS 104-87-0 | E, F | N | Major component of fungal (*Fusarium*) and perfume | Int./Callus: 0.670 Int/Fungus: (-) 0.931 |
| Alkanes | Tetradecane | CAS 629-59-4 | E, F | Y | Major component of perfumes, essential oils | Int./Callus: 0.448  Int/Fungus:0.642 |
|  | Heptadecane | CAS 629-78-7 | E, F | Y | Major component of perfumes, essential oils | Int./Callus: 0.222  Int/Fungus: 0.120 |
|  | Tetradecane, 4,11-dimethyl- | CAS 55045-12-0 | E | N |  | Int./Callus: 1.053  Int/Fungus:0.346 |
|  | Dodecane | CAS 112-40-3 | E, F | Y | Major component of perfumes, essential oils | Int./Callus: 1.126  Int/Fungus:0.794 |
|  | Heneicosane | CAS 629-94-7 | E,F | Y | Major component of perfumes, essential oils | Int./Callus: 0.092  Int/Fungus:0.279 |
|  | Eicosane | CAS 112-95-8 | E, F | Y | Major component of perfumes, essential oils | Int./Callus: 0.399  Int/Fungus:0.616 |
|  | Tetrapentacontane | CAS 5856-66-6 | E,F | N |  | Int./Callus: 0.049  Int/Fungus:1.181 |
|  | 2-methylhexacosane | CAS 1561-02-0 | E, F | N |  | Int./Callus: 0.256  Int/Fungus:0.297 |
|  | Nonane, 4-methyl- | CAS 17301-94-9 | E | N | Major component of perfumes | Int./Callus: 1.850 Int/Fungus: (-) 2.375 |
|  | Dodecane, 2,6,11-trimethyl- | CAS 31295-56-4 | E,F | N | Major component of perfumes | Int./Callus:1.111 Int/Fungus: 0.154 |
|  | Dodecane, 2,6,10-trimethyl- | CAS 3891-98-3 | E, F | Y | Major component of perfumes, agarwood oil | Int./Callus:1.713 Int/Fungus:0.217 |
|  | Heptane, 3,3,5-trimethyl- | CAS 7154-80-5 | E | N |  | Int./Callus: 1.492  Int/Fungus: (-)1.312 |
|  | Cyclopropane, 1,2-dimethyl-3-pentyl-, (1.alpha.,2.alpha.,3.alpha.)- | CAS 62238-10-2 | N | N |  | Int./Callus: 1.641  Int/Fungus:(-)1.465 |
|  | Tetrapentacontane, 1,54-dibromo- | CAS 852228-22-9 | E,F | N | Possibly activated for defence | Int./Callus: 2.499  Int/Fungus: 2.866  (Highest increase) |
|  | Dodecane, 4-methyl- | CAS 6117-97-1 | E | N | Major component of perfumes | Int./Callus:1.202  Int/Fungus: 0.291 |
|  | Decane | CAS 124-18-5 | E,F | N | Major component of perfumes | Int./Callus: 1.99  Int/Fungus: (-) 1.429 |
|  | Cycloundecane, 1,1,2-trimethyl- | CAS 62376-15-2 | E | N |  | Int./Callus: 1.654 Int/Fungus: (-) 1.360 |
|  | Pentadecane, 3-methyl- | CAS 2882-96-4 | E | N | Constituent of aroma profiles | Int./Callus: 0.474 Int/Fungus: 0.457 |
|  | Dodecane, 4,6-dimethyl- | CAS 61141-72-8 | E, F | Y | Agarwood , fragrant | Int./Callus: 1.675  Int/Fungus: (-)2.749 |
|  | Tetracosane | CAS 646-31-1 | E, F | Y | Agarwood, fragrant | Int./Callus: (-) 1.065 Int/Fungus: 1.087 |
|  | Pentatriacontane | CAS 630-07-9 | E, F | Y | Agarwood, fragrant | Int./Callus: 3.471  (2^nd^ highest increase)  Int/Fungus: (-) 0.385 |
|  | 1,1,3,6-tetramethyl-2-(3,6,10,13,14-pentamethyl-3-ethyl-pentadecyl)cyclohexane | Information unavailable | NA | NA | Cyclohexane; fragrant | Int./Callus: 3.635  (Highest increase)  Int/Fungus: (-) 0.627 |
|  | Hexacontane | CAS 7667-80-3 | E | N |  | Int./Callus: (-) 0.795 Int/Fungus: 1.266  (High increase) |
|  | Hexadecane | CAS 544-76-3 | E, F | Y | Major component of perfumes | Int./Callus: 0.767 Int/Fungus: -4.07  (Highest decrease) |
|  | 2,5-Dimethylhexane-2,5-dihydroperoxide  (Hydroperoxide, 1,​1'-​(1,​1,​4,​4-​tetramethyl-​1,​4-​butanediyl)​bis-) | CAS 3025-88-5 | N | N | Defence response: hydroperoxide | Int./Callus: 1.030 Int/Fungus: (-) 3.389 |
|  | Pentadecane, 2,6,10-trimethyl- | CAS 3892-00-0 | E, F | Y | Agarwood, fragrant | Int./Callus: (-) 0.601 Int/Fungus: 0.217 |
|  | Octane, 3,5-dimethyl- | CAS 15869-93-9 | E | N |  | Int./Callus: 1.157 Int/Fungus: (-) 3.630 |
|  | Hydroperoxide, 1-ethylbutyl | CAS 24254-56-6 | E | N |  | Int./Callus: 1.199 Int/Fungus: (-) 1.778 |
|  | Undecane, 4,6-dimethyl- | CAS 17312-82-2 | E, F | N |  | Int./Callus: 1.064 Int/Fungus: (-) 2.362 |
| Aromatics | Benzene, 1,3-bis(1,1-dimethylethyl)- | CAS 1014-60-4 | E, F | N | Fragrant volatile in plants. | Int./Callus: 0.876  Int/Fungus:0.068 |
| Diols | 1,3-Propanediol, 2-butyl-2-ethyl- | CAS 115-84-4 | F | N |  | Int./Callus: 2.185 Int/Fungus(-) 0.542 |
|  | 2,5-Hexanediol, 2,5-dimethyl- | CAS 110-03-2 | E | N |  | Int./Callus: 1.980 Int/Fungus: (-) 1.01 |
| Ketones | Ethanone, 2,2-dimethoxy-1,2-diphenyl- | CAS 24650-42-8 | E,F | N |  | Int./Callus: 1.283 Int/Fungus: (-) 0.816 |
|  | 2-Heptanone, 4,6-dimethyl- | CAS 19549-80-5 | E | N |  | Int./Callus: 1.302 Int/Fungus: (-) 2.350 |
| Terpenoids | Squalene | CAS 111-02-4 | E,F | Y | Major component of terpenoid pathway- perfumes, essential oils; | Int./Callus: (-) 0.011  Int/Fungus: (-) 0.469 |

^1^Search results of SciFinder (E= essential oil/aroma/perfume; F= Fungus including *Fusarium*; Y = Yes; N= No; NA = Information not available in SciFinder).  ^2^ Information from available literature, database, etc.

**Supplementary Table ST -7:** Compounds shared by callus and fungus (i.e. not interaction)

| **Chemical class** | **Name of the compound** | **Chemical identity** | **Reported in essential oil/perfume^1^** | **Reported in agarwood^1^** | **Additional information^2^** |
| --- | --- | --- | --- | --- | --- |
| Acid esters | Propanoic acid, 2,2-dimethyl-, propyl ester | CAS 5129-35-1 | N | N |  |
|  | L-(+)-Ascorbic acid 2,6-dihexadecanoate | CAS 4218-81-9 | A | N |  |
| Alcohols | 1-Heptanol, 2,4-diethyl- | CAS 80192-55-8 | N | N |  |
| Alkanes | Dotriacontane | CAS 544-85-4 | E, F | Y | Agarwood |
|  | Pentadecane, 8-hexyl- | CAS 13475-75-7 | A | Y |  |
| Ketones | 2-Cyclopenten-1-one, 2,3,4,5-tetramethyl- | CAS 54458-61-6 | A | N |  |

^1^Search results of SciFinder (E= essential oil/aroma/perfume; F= Fungus including *Fusarium*; Y = Yes; N= No); ^2^Information from available literature, database, etc.

**Supplementary Table ST- 8: Compounds unique to the profile of callus**

| **Chemical class** | **Name of the compound** | **Chemical identity** | **Reported in essential oil/perfume^1^** | **Reported in agarwood^1^** | **Additional information^2^** |
| --- | --- | --- | --- | --- | --- |
| Acetates | Heptacosyl acetate | ChemSpider ID29739425 | NA | N |  |
|  | 8-Oxabicyclo[3.2.1]octan-3,7-diol, 3-acetate | PubChem CID:538591 | N | N |  |
| Acid ester | Sulfurous acid, 2-propyl undecyl ester | PubChem CID:6420353 | N | N |  |
|  | Docosyl pentafluoropropionate | ChemSpider ID29739951 | NA | NA |  |
|  | Phthalic acid, bis(7-methyloctyl) ester | CAS 20548-62-3 | Y | N | Perfume component |
|  | Carbonic acid, ethyl octadecyl ester | PubChem CID:6421508 | Y | N |  |
|  | Sulfurous acid, butyl heptadecyl ester | CAS 959067-55-1 | Y | N |  |
|  | Benzeneacetic acid, hexadecyl ester | CAS 854646-79-0 | N | N |  |
|  | Hexadecanoic acid, 1-(1-methylethyl)-1,2-ethanediyl ester | CAS 56599-93-0 | N | N |  |
|  | Nonadecyl heptafluorobutyrate  (Butanoic acid, 2,​2,​3,​3,​4,​4,​4-​heptafluoro-​, nonadecyl ester) | CAS 1522155-64-1 | N | N |  |
|  | 4-Fluoro-2-trifluoromethylbenzoic acid, neopentyl ester | ChemSpider ID29749495 | NA | NA |  |
|  | Oxalic acid, 6-ethyloct-3-yl ethyl ester | PubChem CID:6420732 | N | N |  |
|  | Oxalic acid, isobutyl hexadecyl ester | ChemSpider ID4926258 | N | N |  |
|  | Sulfurous acid, octadecyl 2-propyl ester | PubChem CID:6420358 | N | N |  |
|  | Succinic acid, cycloheptyl octadecyl ester | Information unavailable | NA | NA |  |
| Acids | Butanoic acid, 3,3-dimethyl- | CAS 1070-83-3 | Y | N |  |
| Alcohols | 4-Pentene-2-ol, 2-methyl | ChemSpider ID120211 | N | N |  |
|  | 2-Octanol, 3-methyl- | CAS 27644-49-1 | N | N |  |
| Aldehydes | 2-Isopropyl-4-methylhex-2-enal | ChemSpider ID4515502 | Y | N |  |
|  | 3,5-di-tert-Butyl-4-hydroxybenzaldehyde | CAS 1620-98-0 | Y | N |  |
|  | cis-5-Methyl-2-isopropyl-2-hexen-1-al | CAS 69104-97-8 | N | N |  |
| Alkanes | Pentadecane | CAS 629-62-9 | Y | Y | Perfume component |
|  | Cyclooctacosane | CAS 297-24-5 | N | Y |  |
|  | Heptadecane, 2-methyl- | CAS 1560-89-0 | Y | Y |  |
|  | Dodecane, 2-methyl- | CAS 1560-97-0 | Y | N |  |
|  | Octadecane, 1-chloro- | CAS 3386-33-2 | Y | N |  |
|  | Dodecylcyclohexane | CAS 1795-17-1 | N | N |  |
|  | 3,6,6-Trimethylundecane-2,5,10-trione  (2,​5,​10-​Undecanetrione, 3,​6,​6-​trimethyl-) | CAS 959217-13-1 | N | N |  |
|  | Tridecane, 4-methyl- | CAS 26730-12-1 | Y | N |  |
|  | Hexadecane, 4-methyl- | CAS 25117-26-4 | Y | N |  |
|  | Nonadecane, 2,6,10,14-tetramethyl- | CAS 55124-80-6 | N | N |  |
|  | erythro-7,8-Bromochlorodisparlure  (7-Bromo-8-chloro-2-methyloctadecane) | PubChem CID:537616 | NA | NA |  |
|  | Cyclohexane, 1-ethyl-2-propyl- | CAS 62238-33-9 | N | N |  |
|  | Cyclononane, 1,1,4,4,7,7-hexamethyl- | CAS 149331-19-1 | N | N |  |
| Alkenes | 1-Nonadecene | CAS 18435-45-5 | Y | Y | Perfume component |
|  | 3-Octadecene, (E)- | CAS 7206-19-1 | Y | N |  |
|  | Pentalene, octahydro-2-[(2-octyl)decyl]- | CAS 116401-02-6 | N | N |  |
| Aromatics | Benzene, 1,1'-thiobis[2-methyl- | CAS 4537-05-7 | N | N |  |
| Ketones | 1,2:5,6-Di-O-isopropylidene-a-d-ribohexafurnos-3-ulose | PubChem CID:568072 | NA | NA |  |
|  | 4-Isopropyl-1,3-cyclohexanedione | CAS 62831-62-3 | N | N |  |
| Oxiranes | Oxirane, hexadecyl- | CAS 7390-81-0 | Y | N |  |
| Phenols | Phenol, 2,5-bis(1,1-dimethylethyl)- | CAS 5875-45-6 | Y | Y |  |

^1^Search results of SciFinder (Y = Yes; N= No; NA = Information not available in SciFinder).  ^2^ Information from available literature, database, etc.

**Supplementary Table ST-9: Compounds unique to the profile of fungus**

| **Chemical class** | **Name of the compound** | **Chemical identity** | **Reported in essential oil/perfume^1^** | **Reported in agarwood^1^** | **Additional information^2^** |
| --- | --- | --- | --- | --- | --- |
| Acid ester | Oxalic acid, 4-chlorophenyl octyl ester | ChemSpider ID4928219 | N | N |  |
|  | Octadecanoic acid, 17-methyl-, methyl ester | CAS 55124-97-5 | E | N | Esters |
|  | Ethyl Oleate | CAS 111-62-6 | F , E | Y |  |
|  | n-Propyl 9,12-octadecadienoate  (PROPYL LINOLEATE) | CAS 38433-95-3 | E | N | Linoleic acid ester |
|  | 1,2-Benzenedicarboxylic acid, butyl 8-methylnonyl ester  (Phthalic acid, butyl 8-methylnonyl ester) | CAS 89-18-9 | E | N | Perfume component |
|  | Hexadecanoic acid, methyl ester  (Palmitic acid, methyl ester) | CAS 112-39-0 | F,E | Y |  |
|  | Carbamic acid, N-(1,4-dihydro-1-benzyl-4-quinolinylidene)-, ethyl ester | PubChem CID 9602337 | N | N |  |
|  | Nonadecyl pentafluoropropionate | CAS 1578263-27-0 | N | N |  |
|  | (E)-3,7,11-Trimethyldodec-2-enoic acid, methyl ester | PubChem CID: 6422267 | N | N |  |
|  | Tetradecyl trifluoroacetate | CAS 6222-02-2 | E | N |  |
|  | Dotriacontyl heptafluorobutyrate | InChIKey: HDYLMIWYXVQKDS-UHFFFAOYSA-N | NA | NA |  |
|  | 2-Butenedioic acid (Z)-, dibutyl ester | CAS 105-76-0 | F, E | N |  |
|  | Fumaric acid, dodecyl 2-methylallyl ester | Information unavailable | NA | NA |  |
|  | 2-Furancarboxylic acid, dodecyl ester | CAS 116435-27-9 | N | N |  |
|  | Triacontyl pentafluoropropionate | ChemSpider ID29739976 | NA | NA |  |
|  | Oxalic acid, butyl 6-ethyloct-3-yl ester | PubChem CID 6420817 | NA | NA |  |
| Acids | trans-2-Hexadecenoic acid | CAS 929-79-3 | E | Y |  |
|  | Ethyl tridecanoate | CAS 28267-29-0 | F | N |  |
| Alcohols | 1-Decanol, 2-octyl- | CAS 45235-48-1 | F , E | N |  |
|  | 2H-Pyranmethanol, tetrahydro-2,5-dimethyl- | CAS 54004-46-5 | N | N |  |
|  | 1-Octanol, 5,7,7-trimethyl-2-(1,3,3-trimethylbutyl)- | CAS 36400-98-3 | N | N |  |
|  | 11-Dodecen-1-ol, 2,4,6-trimethyl-, (R,R,R)- | CAS 27829-54-5 | N | N |  |
|  | 1-Octacosanol, 2,4,6,8-tetramethyl-, (all-R)- | CAS 27829-63-6 | N | N |  |
|  | 2-Octanol, 2-methyl- | CAS 628-44-4 | E | N |  |
|  | 1-Decanol, 2-ethyl- | CAS 21078-65-9 | F, E | N | Aroma profiles |
| Aldehydes | Cyclopropaneundecanal, 2-nonyl- | CAS 56196-17-9 | N | N |  |
| Alkanes | Nonane, 5-(1-methylpropyl)- | CAS 62185-54-0 | E | N |  |
|  | Tetradecane, 4-methyl- | CAS 25117-24-2 | F, E | N |  |
|  | Nonane, 1-iodo- | CAS 4282-42-2 | F | N |  |
|  | Tridecane, 5-methyl- | CAS 25117-31-1 | F, E | N |  |
|  | Undecane, 3-cyclohexyl- | CAS 13151-78-5 | E | N |  |
|  | Heptacosane | CAS 593-49-7 | F,E | Y |  |
|  | Tridecane, 7-cyclohexyl- | CAS 13151-92-3 | N | Y |  |
|  | Tritetracontane | CAS 7098-21-7 | F, E | N |  |
|  | Decane, 3,6-dimethyl- | CAS 17312-53-7 | F, E | N |  |
|  | Octadecane, 3-ethyl-5-(2-ethylbutyl)- | CAS 55282-12-7 | F, E | Y |  |
|  | Decane, 2-methyl- | CAS 6975-98-0 | F, E | Y |  |
|  | Cyclohexane, 1-methyl-2-pentyl- | CAS 54411-01-7 | N | N |  |
|  | Octane, 6-ethyl-2-methyl- | CAS 62016-19-7 | E | N |  |
| Alkenes | 1-Undecene, 4-methyl- | CAS 74630-39-0 | E | N |  |
|  | 2-Undecene, 4-methyl- | CAS 91695-32-8 | N | N |  |
| Aromatics | Benzene, 1-ethyl-4-methoxy- | CAS 1515-95-3 | F, E | N |  |
| Benzylamine | Dibutylamine, N-benzyl | CAS 4383-27-1 | N | N |  |
| Diols | 2,4,7,9-Tetramethyl-5-decyn-4,7-diol | CAS 126-86-3 | F,E | N |  |
| Ketones | Duroquinone  (2,​5-​Cyclohexadiene-​1,​4-​dione, 2,​3,​5,​6-​tetramethyl-) | CAS 527-17-3 | F, E | N |  |
|  | Z-5-Methyl-6-heneicosen-11-one | PubChem CID 5363254 | N | N |  |
|  | 1,3,7,9-Tetraazaspiro[5.5]undecane-2,8-dione, 3,4,4,9,10,10-hexamethyl- | CAS 73822-04-5 | N | N | Spiro compounds |
|  | Benzophenone | CAS 119-61-9 | F, E | Y |  |
| Nitriles | 2,3-Bis-methylamino-succinonitrile | CAS 18216-45-0 | N | N |  |
| Quinoline | Quinoline, 1,2-dihydro-2,2,4-trimethyl- | CAS 147-47-7 | N | N |  |
| Sulfonyl chloride | 1-Hexadecanesulfonyl chloride | CAS 38775-38-1 | N | N |  |

^1^Search results of SciFinder (E= essential oil/aroma/perfume; F= Fungus including *Fusarium*; Y = Yes; N= No; NA = Information not available in SciFinder).  ^2^ Information from available literature, database, etc.

**Supplementary Table ST-10: Compounds unique to profile of juvenile agarwood plants infected with fungus (H15)**

| **Chemical class** | **Name of the compound** | **Chemical identity** | **Reported in essential oil/perfume^1^** | **Reported in agarwood^1^** | **Additional information^2^** |
| --- | --- | --- | --- | --- | --- |
| Acid esters | Phenacyl hexadecanoate | CAS-41755-61-7 | Y | N |  |
|  | Benzeneacetic acid, 4-pentadecyl ester | PubChem 561138 | NA | NA |  |
|  | Pentanoic acid, 2,2,4-trimethyl-3-carboxyisopropyl, isobutyl ester | IUPAC Standard InChIKey: JIYRKVLLOCBJHR-UHFFFAOYSA-N | NA | NA |  |
|  | Benzeneacetic acid, 3-tridecyl ester | PubChem ID561031 | NA | NA | Ester; perfume component |
|  | Phenylacetic acid, 4-hexadecyl ester | PubChem ID 561161 | NA | NA |  |
|  | Benzyl Benzoate | CAS 120-51-4 | Y | Y | 3 agarwood references |
|  | Isopropyl myristate | CAS-110-27-0 | Y | Y | 1 agarwood reference |
|  | 9,12,15-Octadecatrienoic acid, methyl ester, (Z,Z,Z)- | CAS- 301-00-8 | Y | N | Linolenic acid, methyl ester (6CI,8CI) |
|  | cis-11-Eicosenoic acid, methyl ester | CAS- 2390-09-2 | Y | N |  |
|  | 9-Octadecenoic acid, 1,2,3-propanetriyl ester, (E,E,E)- | CAS - 537-39-3 | Y | N |  |
|  | Phthalic acid, 5-methylhex-2-yl heptadecyl ester | PubChem CID: 91719575 | NA | NA |  |
|  | Phthalic acid, hexadecyl pentyl ester |  | NA | NA |  |
| Acids | Methoprene | CAS 40596-69-8 | Y | N | Juvenile hormone |
| Alcohols | 1-Dodecanol, 3,7,11-trimethyl- | CAS- 6750-34-1 | Y | Y | Agarwood reference; Farnesol- precursor of fragrant compounds; insect pheromone |
|  | 1-Dodecanol, 2-hexyl- | CAS -110225-00-8 | Y | N |  |
|  | 1-Heptatriacontanol | CAS 105794-58-9 | Y | Y | 2 agarwood references |
|  | 9,12,15-Octadecatrien-1-ol, (Z,Z,Z)- | CAS- 506-44-5 | Y | N |  |
| Aldehydes | Undecanal | CAS - 112-44-7 | Y | N | 431 ref of ess oil |
|  | Pentadecanal- | CAS 09-11-2765 | Y | Y | 2 agarwood references |
|  | 9,17-Octadecadienal, (Z)- | CAS- 56554-35-9 | Y | N |  |
| Alkanes | Dodecane, 1-chloro- | CAS- 112-52-7 | Y | N |  |
|  | Hexadecane, 4-methyl- | CAS - 25117-26-4 | Y | N |  |
|  | 2-methyltetracosane | CAS 1560-78-7 | Y | N |  |
|  | Pentatriacontane | CAS 630-07-9 | Y | Y | 2 agarwood references |
|  | Triacontane, 1-bromo- | CAS- 4209-22-7 | Y | N |  |
|  | 2-methylhexacosane | CAS- 1561-02-0 | Y | N |  |
|  | Tetrapentacontane | CAS - 5856-66-6 | Y | N |  |
| Amides | 9-Octadecenamide, (Z)- | CAS- 301-02-0 | Y | N |  |
|  | cis-11-Eicosenamide | CAS- 10436-08-5 | N | N |  |
| Aromatics | Benzene, (1-methylnonyl)- | CAS- 4537-13-7 | Y | N |  |
|  | Benzene, (1-ethylnonyl)- | CAS-4536-87-2 | Y | N |  |
|  | Benzene, (1,2,3-trimethyl-2-cyclopropen-1-yl)- | Cas 6393-13-1 | Y | N |  |
|  | Benzene, (1-methyl-1-propylpentyl)- | CAS 54932-91-1 | Y | N |  |
|  | Pentacosane, 13-phenyl- | CAS- 6006-90-2 | N | N |  |
| Fatty acids | Dodecanoic acid, 2-octyl- | CAS - 40596-46-1 | Y | N |  |
|  | 6-Octadecenoic acid | CAS - 4712-34-9 | N | N |  |
|  | Dodecanoic acid | CAS- 143-07-7 | Y | Y | 2 agarwood references |
| Ketones | 2-Butanone, 4-phenyl- | CAS- 2550-26-7 | Y | Y | 19 agarwood references |
|  | Longipinocarvone | CAS 65556-52-7 | Y | Y | 1 agarwood reference; pheromone |
|  | 2(1H)Naphthalenone, 3,5,6,7,8,8a-hexahydro-4,8a-dimethyl-6-(1-methylethenyl)- | CAS 725240-70-0 | Y | Y | 2 agarwood references |
|  | 6-(1-Hydroxymethylvinyl)-4,8a-dimethyl-3,5,6,7,8,8a-hexahydro-1H-naphthalen-2-one | IUPAC Standard InChIKey: ZQMDPUQDUUCMDK-UHFFFAOYSA-N | NA | NA |  |
|  | Andrographolide | CAS-5508-58-7 | N | Y | 1 agarwood reference |
|  | 2,5-di-tert-Butyl-1,4-benzoquinone | CAS 2460-77-7 | Y | N |  |
| Acetates | 2,6,10,14-Hexadecatetraen-1-ol, 3,7,11,15-tetramethyl-, acetate, (E,E,E)- | CAS- 61691-98-3 | Y | N | Geranylgeraniol acetate; Semiochemical |

^1^Search results of SciFinder (Y= yes; N= No; NA = Information not available in SciFinder). ^2^ Information from available literature, database, etc.
